# Two distinct Okazaki fragment failure modes dominate mutagenesis in the absence of flap endonuclease 1

**DOI:** 10.1038/s41467-026-75267-3

**Published:** 2026-07-17

**Authors:** Scott A. Lujan, Mercedes E. Arana, Hunter Wilkins, Jessica S. Williams, Marta A. Garbacz, Katarzyna Bebenek, Thomas A. Kunkel

**Affiliations:** Genome Integrity and Structural Biology Laboratory, National Institute of Environmental Health Sciences, National Institutes of Health, Research Triangle Park, Research Triable Park, NC USA

**Keywords:** Genomic instability, DNA damage and repair, Mutation

## Abstract

The yeast RAD27 gene encodes the Rad27/Fen1 nuclease, which contributes to genome stability during Okazaki fragment maturation (OFM), DNA mismatch repair (MMR), base excision repair, and other processes. Here, we analyze whole genome mutation accumulation in *Saccharomyces cerevisiae* lacking *RAD27* and show that its loss elevates diverse mutation classes arising through multiple mechanistic pathways. Overall mutation rates in *rad27*Δ cells are over 60-fold higher than in wild type and only modestly lower than in MMR-deficient strains. However, most mutations in *rad27*Δ cells cannot be explained by defective MMR, elevated translesion synthesis, or defects in other Rad27-associated repair processes. Instead, mutation spectra implicate aberrant processing of Okazaki fragment intermediates via DNA terminus slippage before ligation (SBL) and template switching (TS). The latter may arise through replication-associated processes such as transient primer relocation (TPR) or through recombination-mediated mechanisms including homoeologous recombination. SBL accounts for the majority of insertion mutations in *rad27*Δ cells, while TS explains substantial fractions of substitutions, insertions, deletions, and copy number variants, often involving non-local templates. Our results indicate that Rad27 suppresses genome instability through multiple mechanistically distinct roles. Together, these findings reveal Rad27/Fen1 to be a determinant of replication-associated genome stability on par with MMR.

## Abstract (web summary)

Many DNA replication and repair processes require the removal of internal DNA ends. Here, the authors show that loss of the flap endonuclease Rad27/FEN1 causes diverse mutations through two distinct failure modes, revealing a genome stability role for Rad27 on par with that of mismatch repair.

## Introduction

**Fig. 1:**
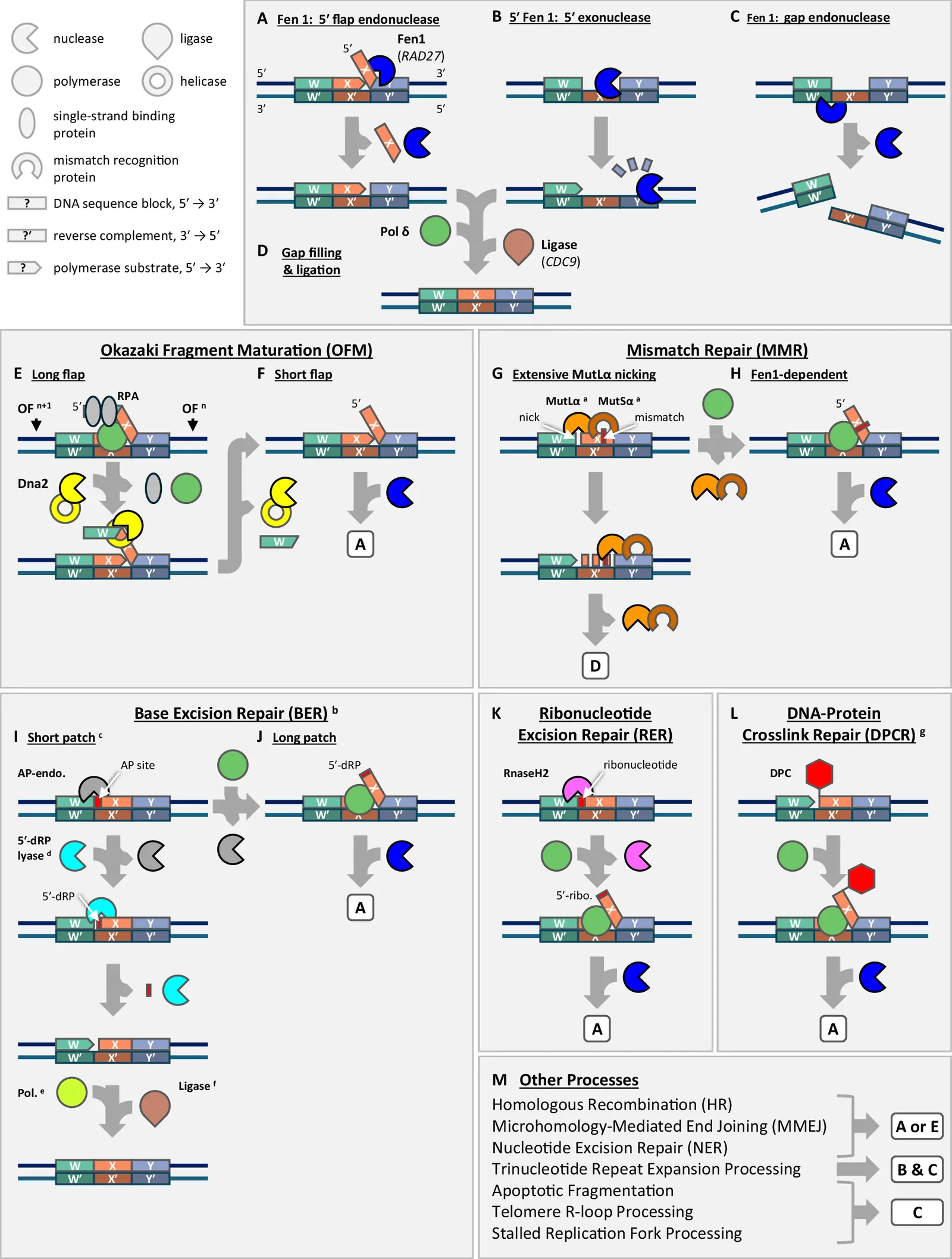
Roles of Rad27/Fen1 in DNA replication and repair. Proposed functions of flap endonuclease 1 (Fen1; Rad27 in Saccharomyces cerevisiae). DNA segments are represented by polygons with inscribed letters; apostrophes indicate reverse complementarity. **A**–**C** Fen1 catalytic activities. Exonuclease 1 (Exo1) provides limited redundancy for some substrates. **A** 5′-flap endonuclease activity. **B** 5′-exonuclease activity. **C** Gap endonuclease activity. **D** DNA synthesis and ligation, a common resolution step for Fen1-dependent pathways. **E**, **F** Okazaki fragment maturation (OFM). Long flaps generated by Pol δ strand-displacement synthesis are coated by RPA and processed by Dna2 (**E**), whereas short flaps are cleaved directly by Fen1 (**F**). **G**, **H** Mismatch repair (MMR). MutSα and MutLα recognize mismatches and direct excision (**G**); strand-displacement synthesis can generate Fen1-dependent flaps (**H**). **I**, **J** Base excision repair (BER). Short-patch BER proceeds through gap filling and ligation without flap formation (**I**), whereas long-patch BER generates a flap removed by Fen1 (**J**). **K** Ribonucleotide excision repair (RER), in which RNase H2 initiates repair and subsequent strand displacement generates a Fen1 substrate. **L** DNA-protein crosslink repair (DPCR), where protein-containing flaps are resistant to exonucleolytic degradation but susceptible to Fen1 cleavage. **M** Additional processes involving Fen1. These include flap processing during homologous recombination (HR), microhomology-mediated end joining (MMEJ), and nucleotide excision repair (NER); suppression of trinucleotide-repeat expansion through removal of aberrant DNA structures; and gap endonuclease-dependent cleavage during apoptotic DNA fragmentation, telomeric R-loop resolution, and replication fork recovery. The strength of evidence for Fen1 in these pathways varies (see citations in text). Notes (see text): a, alternative substrate-dependent MMR complexes; b, vertebrates possess an additional BER pathway; c, some protostomes lack short-patch BER; d-f, repair enzymes vary among organisms; g, DNA-protein crosslinks need not be strand-terminal and the DPCR pathway depicted is human.

The yeast *RAD27* gene encodes Rad27p, also known as Flap Endonuclease 1 (Fen1). Fen1 is a structure-specific nuclease with key roles in diverse replication and repair processes (Fig. 1; reviewed^1^). *RAD27* deletion causes slow growth, hypersensitivity to DNA-damaging agents and increased mutagenesis, including destabilized trinucleotide repeats^2–5^. In mice, heterozygous Fen1 knockout accelerates tumor progression and homozygous knockout causes embryonic lethality^6,7^. Human trinucleotide repeat expansions underlie at least twenty severe neuromuscular and neurodegenerative diseases^8^. Multiple human FEN1 mutations are linked to autoimmunity, chronic inflammation, and cancer^9^. A mutation that disrupts Fen1 interaction with the WRN helicase was identified in a family with non-BRCA hereditary breast cancer and causes aneuploidy-associated tumors in mice^10^. FEN1 activity is also essential for survival of BRCA2-deficient cells^11^. A specific isoform of Fen1^12^ is targeted to the mitochondria^13^, where FEN1 is important for repair of certain oxidized lesions^14^. Rad27/Fen1 activity is thus a central contributor to genome stability and organismal health.

Rad27/Fen1 has three DNA nuclease activities (Fig. 1a–c; reviewed refs. ^15,16^). The 5′-flap endonuclease cleaves 5′ flaps internally at single- to double-stranded junctions (Fig. 1a)^17^. The 5′-exonuclease removes single nucleotides from the 5′ ends of gapped, nicked, or recessed substrates (Fig. 1b)^17–19^. The gap endonuclease cleaves internally at single-stranded gaps or looped DNA structures (Fig. 1c)^15,20^. Such gaps resemble 5′ flaps from the perspective of the enzyme, which recognizes the double-strand/single-strand junction with 5’ ssDNA. In the absence of Fen1, a separate exonuclease, Exonuclease 1 (Exo1), processes 5′ flaps or recessed DNA ends^21^, albeit with reduced efficiency or specificity^22,23^. There is also evidence that the MMR system itself can recognize and remove single-nucleotide Okazaki fragment flaps via MutSα and MutLα-nuclease, respectively^24^. Repair pathways that involve Fen1 converge on a common resolution step involving DNA patch synthesis and ligation (Fig. 1d). Patch synthesis and flap cleavage may occur in any order, but both must precede ligation. In yeast, synthesis and ligation are usually accomplished by DNA Polymerase δ (Pol δ) and DNA Ligase 1 (encoded by *CDC9*), respectively^25^.

About 12,000,000 dNTP incorporation events occur on open single-stranded DNA templates in each haploid *Saccharomyces cerevisiae* nuclear DNA replication cycle. This replication is initiated by synthesis of hybrid RNA-DNA primers by DNA Polymerase (Pol) α-primase followed by extension by Pol δ^26–28^. This is followed by continuous leading strand elongation by Pol ε and discontinuous synthesis of lagging strand segments, Okazaki fragments^29^, by Pols α and δ. Pol δ evidently replicates both DNA strands in termination zones^27^, during break-induced repair^30^, and after replication fork restart^31^. Most replication errors are caught by the intrinsic and extrinsic proofreading activities of Pols ε and δ^32–35^. Most of the rest are corrected by DNA mismatch repair (MMR; Fig. 1g, h), which employs at least two pathways and multiple proteins (e.g. ref. ^24^; reviewed^36–38^). Mismatches are recognized by either MutSα or MutSβ, which recruit MutLα. The latter is an endonuclease which introduces nascent-strand-directed nicks, resulting in short oligonucleotides that can be displaced during patch resynthesis (Fig. 1g)^24,37,39^ or degraded by Exo1 (reviewed^40^). If patch resynthesis causes strand-displacement^41^ the resulting flaps may be removed by Fen1 (Fig. 1h). Comparisons between smaller *rad27*Δ and MMR- data sets have been reported and partial MMR defects attributed to *RAD27* deletion^4,42–44^. Some mismatched substrates may require alternative MMR complexes (e.g., MutLβ, MutLγ), not all of which have endonuclease activity (depending on organism; reviewed^36^), implying assistance in *trans*.

The second most common nuclear DNA transaction during normal replication is Okazaki fragment maturation (OFM), with around 60,000 events per haploid yeast replication cycle. OFM involves dNTP incorporation via strand-displacement synthesis by Pol δ, which displaces the RNA-DNA primer made by Pol α-primase at replication origins and during lagging strand DNA replication. Two pathways have been proposed (reviewed^45–48^). In the short-flap pathway, Fen1 directly removes short flaps produced by Pol δ (Fig. 1f). In the long-flap pathway (Fig. 1e), Pol δ generates flaps ≥20 nt that are coated by RPA^49,50^. RPA is displaced by Dna2 helicase activity and the long flap is shortened by Dna2 endonuclease activity^51^.

Beyond replication, Fen1 participates in multiple repair pathways where strand displacement creates a 5′ flap, including base excision repair (BER, Fig. 1i, j; reviewed^52^). An apurinic/apyrimidinic-site (AP-site) is first created by either spontaneous base loss or removal of a damaged or modified base by a DNA glycosylase. An AP-endonuclease cleaves 5′ to the AP-site, producing a 3′ OH and 5′ deoxyribose phosphate (dRP). In short-patch BER (Fig. 1i), the dRP is removed by a 5′-dRP lyase, and the resulting single-nucleotide gap is filled and ligated without flap formation. Lyase activity is provided by Pols β and λ and other enzymes in vertebrates^53–55^ and by Pol IV and/or Pap2 (Trf4) in *S. cerevisiae*^56–58^. Protostomes which lack Pol β orthologs may rely on an alternative 5′-dRP lyase^59^ or on long-patch BER^56^. Short patch resynthesis is accomplished mainly by X-family Pol β in vertebrates^60^, by the A-family Pol θ in *C. elegans*^59^, and likely by B-family Pol δ in yeast, given no polymerase activity for Pap2 (Trf4) and little effect upon Pol IV deletion^56,61^. Ligation after patch resynthesis occurs by Ligase 1 in mitochondria and in *S. cerevisiae* nuclei^62^, by Ligase 1 and/or Ligase 3 in vertebrate nuclei^63–66^, and possibly by XRCC4, Ligase 4, and XLF in *Drosophila melanogaster*^67^. In long-patch BER (Fig. 1j), required for lesions such as 2-deoxyribonolactone in mitochondria^14^, strand displacement generates a flap that Fen1 cleaves^68^. Vertebrates have a third BER pathway involving RECQ1, ERCC1-XPF, PARP1 and RPA, with flap removal proceeding as per long-flap OFM^69^.

Fen1 has also been implicated in many other genome-maintenance processes (Fig. 1k–m). In ribonucleotide excision repair (RER), RNase H2 nicks 5′ to an embedded ribonucleotide, creating a 3′ DNA end (Fig. 1k). Flap intermediates formed by subsequent strand displacement are resolved by Fen1^23,70^. In DNA-protein crosslink repair (DPCR; Fig. 1l), strand-displacement synthesis produces flaps which contain a covalently attached protein (e.g. Topo IIIa) or a protein fragment. These bulky adducts render the flap resistant to 5′ exonucleolytic degradation but susceptible to Fen1 endonuclease^71^, which is recruited by PARP in humans^72^. Homologous recombination (HR)^16^, microhomology-mediated end joining (MMEJ)^11,16,73^, and nucleotide excision repair (NER)^74,75^ all generate flaps that may be processed by Fen1, with or without Dna2, depending on flap length and RPA binding (Fig. 1m; strength of evidence varies). Trinucleotide repeat expansions are suppressed by Fen1 exonuclease and gap endonuclease removal of aberrant secondary structures formed during replication^5,76^. Apoptotic DNA fragmentation^15,77^, telomeric R-loop resolution^16,78,79^, and replication fork recovery^15,20,80–82^ all involve cleavage at internal or branched DNA structures via the gap endonuclease activity of Fen1^75^.

Here we report the consequences of deleting the *Saccharomyces cerevisiae RAD27* gene on mutations that accumulate in the nuclear genome after a defined number of DNA replication cycles. The results reveal that Rad27 is likely involved in preventing several types of genome instability that arise by the mechanisms just described.

## Results

**Fig. 2:**
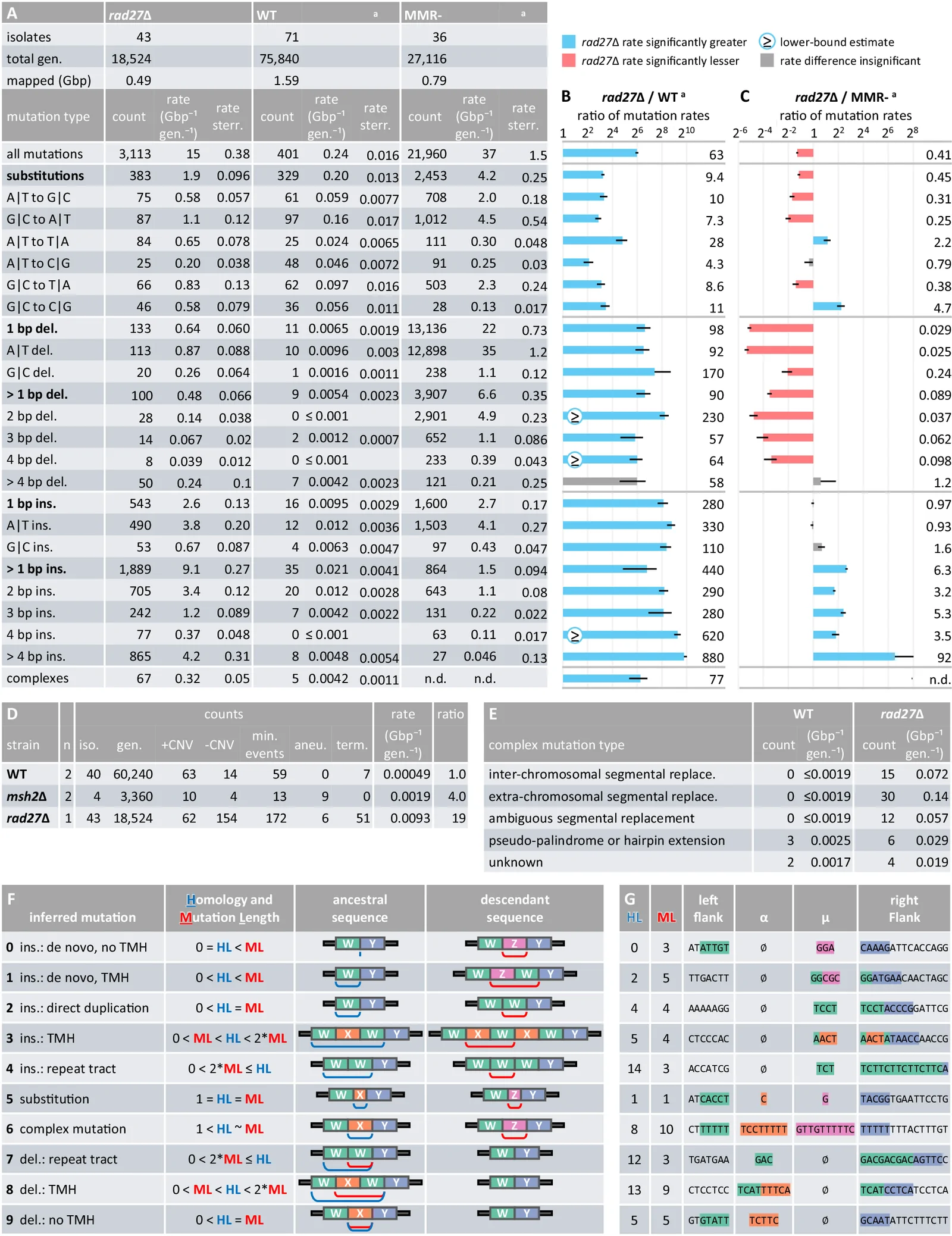
Whole genome mutation rates in wild type, *rad27*Δ, and MMR- strains. Mutation rates were determined from whole-genome mutation accumulation experiments in *Saccharomyces cerevisiae*. **A** Mutation counts and rates. The sum of counts in subcategories may be less than the count in an overarching category due to mutations that are difficult to subcategorize. The sum of rates in subcategories may be greater than the rate in an overarching category when subcategories have lower target counts (e.g. only A/T loci vs. all loci). Notes: gen., generations; sterr., standard error; a, data from L03, S288c, and W303 yeast backgrounds were combined here, only L03 was used for Fig. 3. **B** Ratio of *rad27*Δ and WT mutation rates. *RAD27* deletion significantly increased nearly all mutation classes (blue bars; Welch’s *t* test, *p* < 0.0015 after Šidák correction). Error bars indicate standard errors of the ratios. Where no WT mutations were observed, lower-bound ratios were estimated assuming a single WT event (≥). **C** As per (**B**), but for ratios of *rad27*Δ and MMR− mutation rates. Many mutation rates were lower in *rad27*Δ than in MMR− strains (red bars), whereas rates elevated in *rad27*Δ (blue bars) cannot be explained solely by loss of mismatch repair. **D** Copy-number variation (CNV) in selected strains. Rates are based on minimum inferred event counts. Aneuploidy events involve whole chromosomes; terminal CNVs involve chromosome ends. Notes: +CNV, increase in copy number; −CNV in copy number; term., terminal CNV, including chromosome end; n, ploidy; iso., isolates; gen., generations. **E** Complex mutations. Complex events were classified as segmental replacements when the mutant sequence and significant flanking sequence matched another genomic locus. Replacements were further categorized as inter-chromosomal, extra-chromosomal, or ambiguous depending on the location of the best-matching donor sequence. Shorter mutations require longer matching flanks to establish significance. **F** Mutation classification by mutation length (ML) and local sequence homology length (HL). Notes: TMH, terminal microhomology; α, reference sequence; μ, mutant sequence; ∅, absence of base pairs. **G** Representative examples of each mutation category shown in panel F. Example source, from top to bottom (sample/chromosome/position): TAK2559/11/597,235; TAK2557/14/428,109; TAK2600/4/645,071; TAK2558/14/347,240; TAK2557/14/626,725; TAK2557/3/249,784; TAK2560/7/611,566; TAK2559/9/382,109; TAK2566/7/529,970; and TAK2564/16/729,102. Source data are provided as a Source Data file.

We assessed the role of the Rad27/Fen1 nuclease in modulating nuclear genome stability using a whole-genome mutation accumulation approach. This was done by passing 43 independent clones of a haploid *rad27*∆ strain through fifteen single-cell bottlenecks on solid media (forked lineages reduced the effective mean to 14.4 passages). Samples were collected from ancestral populations prior to the experiment, allowing comparison of ancestral and descendant genome sequences for mutation calling via the *muver* pipeline^83^. A total of 3113 point mutations and 172 copy number variations (CNVs) accumulated over a collective of >18,000 total yeast generations (Fig. 2A and Supplementary File 1). We used these mutations to calculate mutation rates per billion base pairs per generation (Gbp⁻¹ gen.⁻¹). We then compared these rates to rates in wild type and mismatch repair-deficient strains (MMR-; Table 1)^33,83–86^.

**Table 1:** Whole genome mutation accumulation (WGS MA) parameters by strain

				Counts			Mapped	rate		WGS MA
Set	Strain	Background	Ploidy	Isolates	Generations	Mutations	(Gbp)	(Gbp⁻¹ generation⁻¹)	Standard deviation	Citation
WT	*wt*	Δ7	2	39	53,760	247	0.867	0.20	0.073	^33^
	*wt*	W303	2	24	14,880	102	0.533	0.32	0.16	Here
	*wt*	S288C	2	8	7200	56	0.186	0.34	0.16	Here
MMR-	*msh2*Δ	Δ7	2	5	4260	4010	0.106	43	8.5	^83,85^
	*pms1-DE*	Δ7	2	8	6420	4549	0.174	35	17	^86^
	*msh2*Δ	S288C	2	8	4032	3494	0.177	39	3.3	Here
	*mlh1*Δ	Δ7	2	15	12,404	9913	0.336	36	2.7	^84^
*rad27*Δ	*rad27*Δ	Δ7	1	43	18,542	3113	0.487	15	2.5	Here
*rad27*Δ dip.	*rad27*Δ	Δ7	2	22	3960	776	0.492	8.8	0.46	Here

Perhaps unsurprisingly given the number of pathways in which Rad27 participates (Fig. 1), the *rad27*∆ strain yielded the most complicated whole-genome spectrum that we have ever collected in a yeast whole-genome mutation accumulation experiment. Among the point mutations, 97.8% were substitutions, insertions, or deletions. The remaining 2.2% (67 mutations) were complex clusters of sequence changes at positions separated by fewer than twelve base pairs. Another 70 variants were caused by combining point mutations with CNVs, discussed below regarding loss of heterozygosity (LOH). Another 12 variants were attributed to subclonal insertions in ancestral samples. These 82 variants are not included in mutation counts but may be found in Supplemental File 1.

The total nuclear mutation rate upon deleting *RAD27* is 15 Gbp⁻¹ gen.⁻¹ (Fig. 2A). This rate is 63-fold *higher* than in wild type cells (0.24 Gbp⁻¹ gen.⁻¹; Fig. 2B) and 2.4-fold *lower* than in MMR- cells (37 Gbp⁻¹ gen.⁻¹; Fig. 2C). The *rad27*∆ rate is 2.3-times the rate reported in a smaller *rad27*Δ survey^87^, a significant difference (*p* < 10^−9^). We attribute this difference to mutation calling sensitivity, because our *muver* pipeline is especially adept at calling multi-base indels from mutation accumulation experiments^83^. In all three strain sets, mutations are distributed across all 16 nuclear chromosomes (Supplementary Fig. 1; Supplementary Figs. S2, 3 for specific mutation types in the *rad27*Δ strain). Thus, the results indicate that Rad27 is very important for maintaining stability across the yeast nuclear genome.

### Deleting *RAD27* increases base substitution mutation rates

The *rad27*∆ mutant increases the overall single base substitution rate across the nuclear genome by 9.4-fold (Fig. 2B). This is just under half the increase seen for total loss of MMR (Fig. 2C). Among the six types of single base substitutions, the increases due to loss of Rad27p range from 4.3-fold for A•T to C•G substitutions up to 28-fold for A•T to T•A substitutions (Fig. 2B). Substitution rates are similar to those seen in one earlier study^42^ and somewhat larger than in an another^87^. These smaller studies may be different due to differences in strain background and/or mutation calling pipelines. Elevated *rad27*∆ substitution rates could conceivably be explained by three nonexclusive mutagenic routes: 1) loss of MMR efficiency; 2) loss of damage repair efficiency leading to unrepaired lesions or increased error-prone translesion synthesis; and/or 3) aberrant processing of unprocessed DNA flaps in the *rad27*∆ strain.

Substitution rates in *rad27*∆ cells are 45% of the rates in MMR- cells (Fig. 2C). These lower total rates are statistically significant (*p* < 10^−12^) and are primarily due to lower rates for the transitions (*p* < 10^−13^). In contrast, A•T to T•A and G•C to C•G transversion rates in the *rad27*∆ strain exceed those in MMR- strains by 2.2 and 4.7-fold (*p* = 1.6 × 10^−4^ and 6.3 × 10^−7^, respectively), while A•T to C•G rates do not differ significantly (*p* = 0.12).

### Deleting *RAD27* strongly increases single base deletion rates

A variety of indel rates are elevated in a variety of sequence contexts in the *rad27*∆ strain. Among these are numerous single-base deletions of both A•T and G•C base pairs that are observed in the *rad27*∆ strain (Fig. 2a). The overall rate for deletion of a single base pair in the *rad27*∆ strain is 0.64 ± 0.060 Gbp⁻¹ gen.⁻¹. This is 98-fold greater than in wild type yeast (Fig. 2B) but is 34-fold less than in the MMR- strains (Fig. 2C). Greater than 83% of these events in the *rad27*∆ strain (111 of 133) are observed within homonucleotide sequences. Such single base deletions result from mismatches consisting of an extra base in the template strand.

The rates for loss of individual A or T and G or C bases are 92-fold and 170-fold higher, respectively, in the *rad27*∆ strain as compared to wild type yeast (Fig. 2B). In *S. cerevisiae*, A•T base pairs outnumber G•C base pairs (62% to 38%) and A•T mononucleotide runs are more numerous, especially for longer run lengths^88^. After accounting for base pair abundance, the A•T deletion rate is significantly higher than for the G•C deletion rate (3.5-fold; 0.87 ± 0.088 versus 0.26 ± 0.064 Gbp⁻¹ gen.⁻¹; *p* = 5.1 × 10^−7^; Fig. 2B). This imbalance is in the same direction, but of lower magnitude, than in wild type and MMR- strains (6.1 and 33-fold, respectively; *p* < 2.8 × 10^−2^ and *p* < 10^−20^, respectively).

### Single-base additions are elevated in the *rad27*∆ strain

The single base addition rate is elevated in the *rad27*∆ mutant by 270-fold over the rate in the wild type strain (0 .0095 ± 0.0027 versus 2.6 ± 0.13 Gbp⁻¹ gen.⁻¹; Fig. 2A, B). This increase is equivalent to that observed in MMR- strains (2.7 ± 0.13 Gbp⁻¹ gen.⁻¹; Fig. 2a, c). The single base addition rate in the *rad27*∆ mutant is 4-fold *higher* than the single base deletion rate in this same strain. This differs from the MMR- strains, wherein the single base deletion rate is 8-fold *greater* than the addition rate (Fig. 2A).

### Two to four base indel rates are preferentially increased in the *rad27*∆ strain

Included among the mutation types in the *rad27*∆ strain are deletions and insertions of two to four base pairs. Relative to rates in wild type strains, these are 64- to 880-fold more frequent in the *rad27*∆ strain, depending on indel type and length (Fig. 2B). As in the MMR- strains, for a given indel type, indel rates generally decrease with indel size. This is predicted by the increasing number of hydrogen-bonded base pairs that must be disrupted in order for DNA strand slippage to occur during replication^89^.

The two-to-four base deletion rate in the *rad27*∆ strain is lower than in MMR- strains (red in Fig. 2c), but insertion rates are higher for all lengths greater than 1 bp (blue in Fig. 2c). Indeed, rates for longer additions (>4 bp) are 92-fold higher in the *rad27*∆ strain than in MMR- strains. Large increases in multi-base addition rates were noted before for *rad27*∆ strains^87^, including duplications of up to 100 bp ^4^, and were attributed to failure to remove DNA flaps.

78% of deletions in *rad27*∆ cells, including 79% of class 7–8 deletions (Fig. 2f), fall outside of the 2–5 bp range. Only 3 of 28 *rad27*∆ dinucleotide deletions are found in AC or AG repeats (see discussion of RER contributions).

### Deleting *RAD27* increases the rate of copy number changes

Also observed in the *rad27*∆ strain were large-scale changes to copy number that imply very large indels and/or genomic rearrangements (Fig. 2D). The *rad27*∆ strain used here is globally haploid, but the sequencing depth was higher than anticipated in some loci (e.g. *RUF1* to *CUP2*) even before mutation accumulation began. This is normal with the L03 reference sequence and is accounted for by comparing sequences from initial and final samples so that only copy number variation (CNV) events that occurred during the experiment are counted toward the rate. In the 43 outgrowth lines, various loci increased by 62 copies relative to the initial samples, while other loci decreased by 154 copies. Local copy number may change in small increments over time, or if all copies are on one chromosome, it may as much as double or drop to zero in a single event. Given the rarity of copy number changes, the principle of maximum parsimony was applied here, yielding a minimum of 172 events and a rate of at least 0.0093 Gbp⁻¹ gen.⁻¹. This is 19-fold higher than the rate in wild type cells (0.00049 Gbp⁻¹ gen.⁻¹), and it is 4.9-fold higher than the rate in *msh2*Δ cells (0.0019 Gbp⁻¹ gen.⁻¹).

CNV locations differed between strains, with 9 of 13 in the *msh2*Δ strain involving entire chromosomes (i.e. aneuploidy), as compared to 6 of 172 in the *rad27*Δ strain and 0 of 59 in the wild-type strain. In contrast, 51 of 172 CNV event in the *rad27*Δ strain were inferred to involved ends of chromosomes (see Methods), compared to 7 of 59 in the wild- type strain and none in the *msh2*Δ strain. In both *msh2*Δ and *rad27*∆ strains the remaining CNVs, those not involved in aneuploidy or chromosome ends, terminate on homologous loci. These were usually pairs of transposons, orphaned LTRs, or paralogous genes (Supplementary File 1).

### Deleting *RAD27* increases the rate of complex mutations

Complex mutations were called with high confidence in *rad27*Δ and in the Δ7 wild type strains (Fig. 2e). Complex mutations were categorized as hairpin/pseudo-palindrome extensions or as segmental replacements. Segmental replacements were further classified as inter-chromosomal conversions (ICCs), extra-chromosomal conversions (ECCs), or ambiguous conversions depending on whether the best-matching donor sequence occurred on the same chromosome, different chromosome(s), or both. Complex mutations that matched no other loci in the genome with sufficient significance were classified as unknown.

Only five complex mutations were identified in the wild type cells after over 60 thousand generations of accumulation, yielding a rate of 0.0042 ± 0.0019 Gbp⁻¹ gen.⁻¹. *RAD27* deletion elevated the rate 77-fold, to 0.32 ± 0.050 Gbp⁻¹ gen.⁻¹. Three of the five observed complex mutations in wild type cells extended or created hairpin/pseudo-palindrome sequences. In contrast, the 67 complex mutations observed in the *rad27*Δ strain included only six such extensions. Instead, 57 of the *rad27*Δ complexes were segmental replacements. Thirty of these are ECCs, 15 are ICCs, and 12 are ambiguous conversions. This contrasts with previous results, in which the spectrum of complex mutations was dominated by hairpin extensions and very local segmental replacements^87^.

In the *rad27*Δ strain, on average, the smallest complex mutations were from the unknown category, which had mutation and reference sequence lengths of 1–3 base pairs (AGA → GGG; T → GA; TCG → ACT; and G → CT). The longest complex examples were ICCs, roughly twice as long as ECCs and ambiguous conversions, on average, though the differences were not significant (ICC:ECC:ambiguous; 10.7 ± 5.1:4.7 ± 2.7:5.2 ± 3.7 bp reference; 10.1 ± 5.8:4.6 ± 2.4:5.0 ± 2.6 bp mutant; mean ±1 standard deviation).

Mutant sequences of all ECCs and ICCs matched a target sequence elsewhere in the genome, plus at least four base pairs of identity on at least one flank. On the opposite flank, all but one case also had at least a single matching base pair. The lone exception was an ICC on chromosome 11 (TTTTGACATATTT → aaTTTctAgcTtTTaa, position 342,254, sample TAK2600), which had five upstream bases of identity but none downstream. The ambiguous conversions likewise showed at least four base pairs of identity on one flank and no more than one base pair on the other flank. In several cases, flanking identity extended beyond the 10 bp search window (some manually extended; Supplementary File 1). These minimal flanking requirements point to mechanistic constraints.

Of the 11 *rad27*Δ complex mutations that could be classified as tandem substitutions, five created CC dinucleotides and none originated from GC or TC dinucleotides. All could be attributed to either conversions or hairpin/pseudo-palindrome extension. See the Discussion for a comparison to Pol ζ mutation spectra.

### Redefining mutation in terms of length and sequence context

The four point mutation types (substitutions, insertions, deletions, complex mutations) have been traditionally separated into ten classes based on the degree of local homology (Fig. 2f). Categories 0–4 are insertions: (0) new sequence with no local homology; (1) new sequence with at least one base of terminal microhomology (TMH); (2) direct duplication of a non-iterated sequence block; (3) direct duplication of a sequence block with terminal microhomology; and (4) insertion of a repeat unit into a repetitive tract. Categories 5–6 are segmental replacements: (5) single-base substitutions and (6) complex mutations. The latter may involve replacement with a sequence found elsewhere in the genome or by synthesis of a wholly new sequence. Categories 7–9 are deletions: (7) deletion of a repeat unit from a repetitive tract; (8) deletion of a sequence block with terminal microhomology; and (9) deletion of a sequence block with no local homology. Mutations of all categories were observed in *rad27*Δ outgrowths (examples in Fig. 2g; counts and discussion below; full list in Supplementary File 1).

### Deleting *RAD27* preferentially increases indel rates in specific genomic regions

**Fig. 3:**
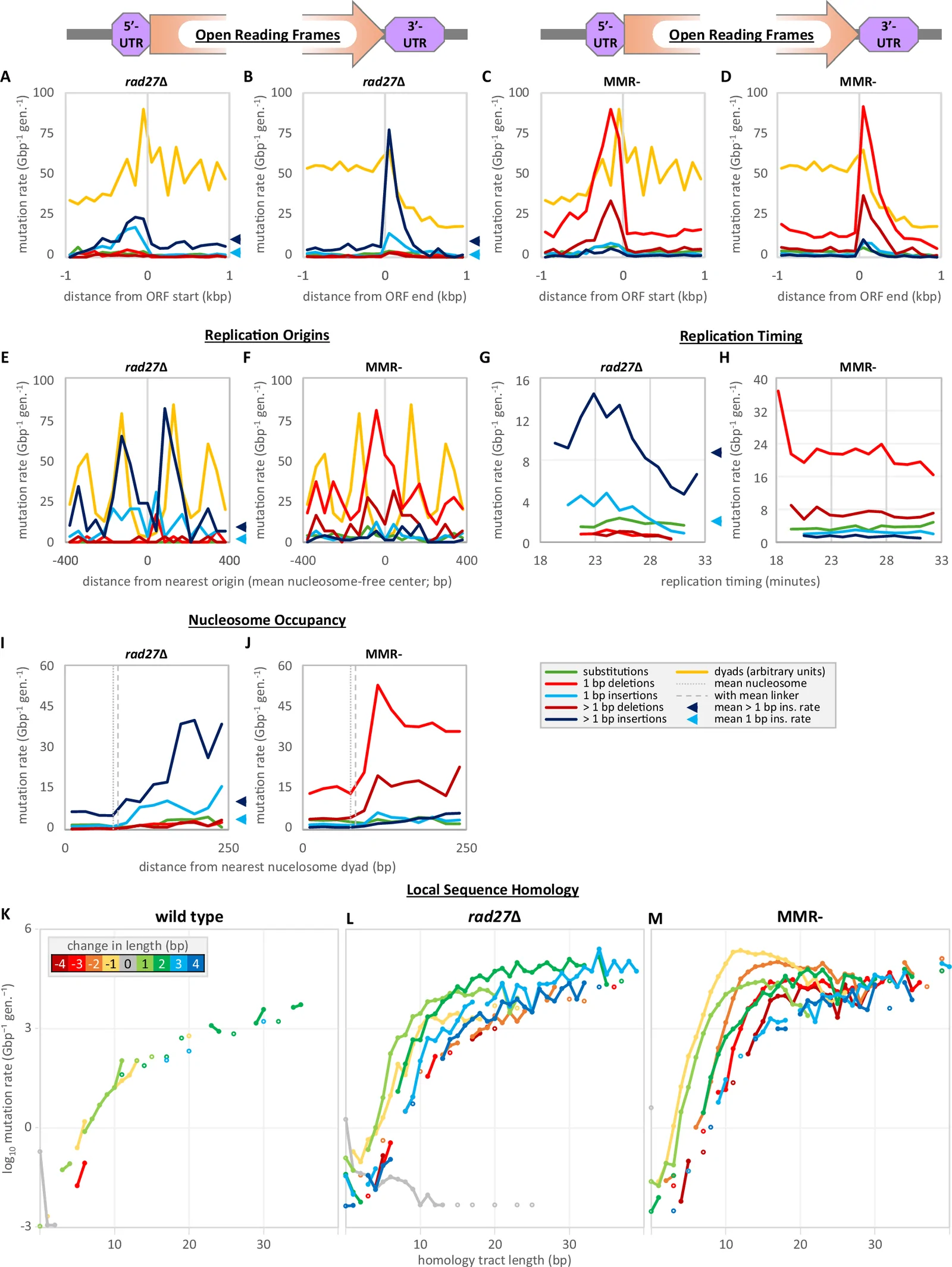
Mutation rates and nucleosome densities by locus. Curves are colored by mutation type with matching arrows indicating mean rates. Nucleosome positions were mapped in the Δ7 strain background previously^85^. Only samples with the Δ7 strain background were used in panels (**A**–**J**). Dyad densities are scaled to fit each plot (orange curves). **A**–**D** Mutation rates and nucleosome densities around open reading frames (ORFs). **A** Mutation rates around ORF starting positions in the *rad27*Δ strain. Each mutation is assigned to the nearest ORF starting position only, to avoid double counting, and the rate scaled to the target size in each bin. Therefore, negative positions represent only extragenic sequences and positive positions represent coding sequences. **B** As per (**A**), but for ORF end positions. Negative and positive positions represent extragenic and coding sequences, respectively. **C**, **D** As per (**A**, **B**), but for the MMR- strains. Mutation and nucleosome densities around replication origins in the (**E**) *rad27*Δ and (**F**) MMR- strains. When replication origin consensus sequences (ACSs) were aligned, nucleosome densities were symmetrical about a point ~25 bp downstream (3′ direction) from the start of the ACS. This point was set to 0. **G**, **H** Rates as per (**E**, **F**) but binned by replication timing. The time, after release from cell cycle arrest, was measured previously^129^ and mapped onto the L03 reference sequence^85^. **I**, **J** As per (**E**, **F**) but for distance from nucleosome dyads. Nucleosome bound DNA (dotted gray line) and the nucleosome size with the mean linker (dashed gray line) are indicated. **K**–**M** Mutation rates, per base pair, versus homology tract length, colored by the resulting change in sequence length. **A** Rates in wild type strains. **B** Rates in rad27Δ cells. **C** Rates in MMR- strains. Source data are provided as a Source Data file.

Insertion rates in the *rad27*∆ strain and deletion rates in MMR- strains peak just outside of the first and last nucleosomes of open reading frames (ORFs; Fig. 3a–d). These regions are enriched for repeat tracts, especially long A•T homonucleotide runs^85^. Likewise, insertion rates in the *rad27*∆ strain and deletion rates in MMR- strains peak in the linkers between adjacent nucleosomes (ORFs; Fig. 3i, j). These regions are also enriched for long A•T homonucleotide runs, which disfavor yeast nucleosome binding (ref. ^90^ and reviewed in refs. ^91,92^).

Replication origins are also enriched in A•T homonucleotide runs, so it is unsurprising that deletion rates in MMR- cells also peak there (Fig. 3f). In contrast, there are two multi-base insertion peaks flanking the origin in the *rad27*∆ strain (Fig. 3e). The peaks are approximately symmetrical about the origin midpoint, defined here as the point approximately 25 bp downstream (3′ direction) from the beginning of the replication origin consensus sequences (ACSs). Overall, multi-base insertion rates around origins in the *rad27*Δ strain appear periodic with a period of ~210 bp.

### Deleting *RAD27* preferentially increases mutation rates early in replication

Substitution rates in *rad27*∆ cells did not deviate from uniformity with respect to replication time (Fig. 3g; nonuniformity *p* = 0.18), just like rates for all mutation types in MMR- cells (excluding 1 bp deletion in the earliest timepoint, representing the earliest 1.1% of the genome; Fig. 3h). In contrast, *rad27*∆ rates for each indel category decrease between early and late replication (early maximum to late minimum: 3-fold each for multi-base insertions and deletions; 5-fold each for single-base insertions and deletions). Insertion rates were highly nonuniform (Fig. 3g; *p* < 10^−24^). Multi-base insertion rates peaked at 14 Gbp^−1^ gen.^−1^ by 23 min after release and decreased to 4.7 Gbp^−1^ gen.^−1^ by 31 min. The mechanism behind these decreases is unknown (see Discussion).

### Deleting *RAD27* preferentially increases indel rates at loci with high local homology

We have discussed mutations by size, but mutations may also be classified by the extent of local sequence homology. We have shown indel rates by tract length in a variety of strains^32,83,93–95^, including many of the MMR- strains used here^88,96^. Here we show a variation on such plots where the horizontal axis is the extent of local sequence homology (Fig. 3k–m). The extent of local sequence homology is the number of consecutive bases in the original sequence that match the terminal sequence of the mutation (see Methods for mathematical expression). It is rarely known which repeat unit in a tract was unpaired during the mutagenic process that created an indel. Local sequence homology length is thus calculated by looking for consecutive, flanking repeats of the mutation sequence, including partial repeats. The mutation sequence is effectively wrapped into a circle and rolled along the flanking sequence until a mismatch occurs. The distance rolled is the local sequence homology length.

We illustrate here with two examples. Example 1 is a single-base deletion in a run of seven Ts. The mutation caller is presented with a six bp run in a descendant sample and a seven bp run at the corresponding ancestral locus. The single deleted T matches each base in the run. Therefore, there are seven bp of local sequence homology and a 1 bp decrease in chromosome length. Example 2 is an ancestral sample with a 5′-ATTTA sequence (5 bp) at an outgrowth locus with the sequence 5′-ATTTATTTA. This is a 4 bp insertion of either ATTT or TTTA. Either way, the ancestral sequence represents 1.25 consecutive copies of the inserted sequence, a 4 bp increase in chromosome length with 5 bp of local homology.

Substitution mutations do not change chromosome length (0 bp change) and only depend on extended homology in certain cases^86^. Some complex mutations do change chromosome length, but they are defined here as 0 bp changes for display purposes. Here, for the sake of simplicity, local homology for substitutions and complex mutations is set to 0 bp.

Figure 3k–m depict mutation rates by local homology length, with curves colored by the mutational change in chromosome length. Deletions are negative changes in length and insertions are positive. Rates are calculated, as always, by dividing the mutation count by the target count and then by the number of elapsed generations. Target counts include all loci wherein a given mutation could conceivably occur. For previous Examples 1 and 2, this would be, respectively, all 7 bp homopolymers and all 5 bp sequences with exactly one identical base on each terminus. See the Methods for counting algorithms.

As with previous MMR- experiments^88,96^, indel rates vary widely with homology tract length. Indel rates in tracts of at least 10 bp are at least 1000-fold higher than rates for indels of the same size in 5 bp tracts. The most extreme difference is for single-base deletions in the MMR- strains, where homology tracts for single-base are, by definition, homopolymers. The single-base MMR- deletion rate is 9.4 million-fold higher in 12 bp runs than in 1 bp runs, i.e. non-iterated bases (230,000 versus 0.024 Gbp⁻¹ gen.⁻¹, respectively). These rates are per base pair, rather than per tract. The rate per tract is the per base rate times the length of the tract, so the rate in 12 bp runs is 110 million-fold higher than for 1 bp runs. In MMR- cells, rates per tract eventually peak or plateau at tract lengths above 10 bp, earlier for smaller indels than for larger indels. Rates that increase with tract length were predicted energetically, with positions of peaks and plateaus attributed to the contact footprint of the replicative polymerase and proofreading exonuclease domains^88^. Overwhelmingly higher indel rates in long tracts are sufficient to explain rates that peak in repeat-dense untranslated regions around ORFs (Fig. 3a–d), at replication origins (Fig. 3f) and in long linkers between nucleosomes (Fig. 3i, j). They explain neither the twin multi-base insertion peaks at origins (Fig. 3e) nor higher indel rates in early replication (Fig. 3g) seen in *rad27*Δ yeast.

Indel rates are lower and increase more slowly with homology tract length in wild type cells (Fig. 3k) than in MMR- cells (Fig. 3m), but *rad27*Δ indel rates are more complicated (Fig. 3l). In tracts over 7 bp, *rad27*Δ deletion and single-base insertion rates are intermediate between wild type and MMR- rates. Multi-base insertion rates are higher in *rad27*Δ cells than in MMR- cells but single-base insertion rates in short tracts (<7 bp) are broadly similar. Note that *rad27*Δ segmental replacements (substitutions and complex mutations) generally decrease in rate with increasing length (Fig. 3l, gray curve).

### The effects of *RAD27* deletion are sequence context dependent

**Fig. 4:**
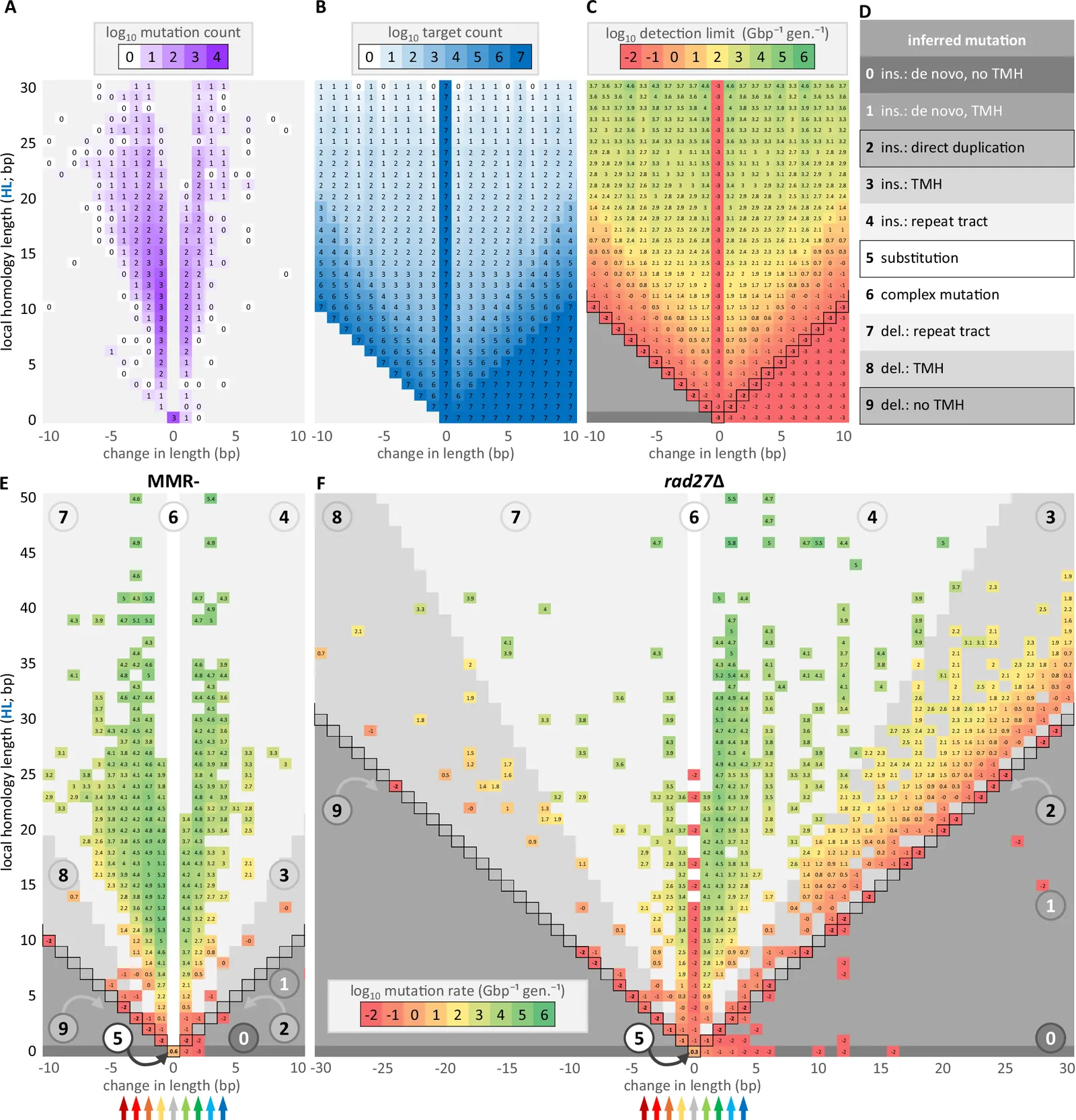
Fountain plots relate mutation rates to mutation size and local sequence homology. **A**–**C** How fountain plots are calculated (example data from MMR- strains). **A** Mutation counts. **B** Target counts, i.e. the number of loci where a mutation length and local homology length are both possible. Rates are calculated by dividing mutation counts by target counts and by the number of elapsed generations. **C** Detection limits. These are the minimum mutation rates calculable from one observed mutation. **D** Mutation categories, colored to match their corresponding fountain plot sections. **E**, **F** Fountain plots, i.e heatmaps depicting mutation rates as a function of mutation length and local sequence homology. Rates are in log_10_, per Gbp per generation. Arrows beneath fountain plots indicate data columns used to build curves in Fig. 3l, m, colored to match those curves. Plots are divided into twelve sections with ten representing the possible mutations defined in panel (**D**). Deletions lie to the left (negative length changes), insertions to the right, substitutions at the origin, and complex mutations along the y-axis. **E** Fountain plot for the MMR- strains. High mutation densities in individual samples precluded confident complex mutation calling. 89.5% of point mutations are within the depicted area. **F** Fountain plot for the *rad27*Δ strain. 95% of point mutations are within the depicted area. Source data are provided as a Source Data file.

If all ten mutation categories presented in Fig. 2f can be defined by mutation length and local homology length, then their rates can be visualized on one plot. Here we introduce fountain plots, heatmaps designed for this holistic assessment of mutation rates (Fig. 4). These were named for their resemblance to multiple fluid streams erupting from the origin. Rates are calculated, as always, by dividing the mutation count (Fig. 4a) by the target count (Fig. 4b) and then by the number of elapsed generations. As such, the minimum measurable rate (detection limit; Fig. 4c) decreases as target count or accumulation period increase (e.g. increase ploidy, outgrowth count, or generation count). Log scale rates, indicated by a color gradient, are plotted against the change in chromosome length due to a mutation (horizontal axis; as represented by curve colors in Fig. 3k–m) and the number of base pairs of local sequence homology (vertical axis; as per Fig. 3k–m horizontal axes). The data underlying depicting curves in Fig. 3k–m are represented by the columns indicated by color-coded arrows below Fig. 4e, f. Each fountain plot is divided into twelve sections (Fig. 4d), ten of which are numbered and shaded to match the mutation classes previously defined (Fig. 2f).

Fountain plots for MMR- and *rad27*∆ strains differ in several interesting ways. 87% of MMR- indels are deletions while 91% of *rad27*∆ indels are insertions. Indels in MMR- strains are also largely confined to repeat tracts, in sectors 4 and 7, with only 0.29% of indels (56 of 19,508) outside of those (Fig. 4e). The MMR- distributions make sense for errors made primarily during bulk synthesis by replicative polymerases, where deletions are lower energy than insertions and slippage in repeat tracts is common. In contrast, *rad27*∆ indels are more evenly distributed across the eight indel classes, with 33.5% (891 of 2662) outside of classes 4 and 7 (Fig. 4f). Most of these are insertions with TMH in sector 3, which have been attributed to flap re-equilibration^87^.

In some areas, mutation rates form smooth continua across class boundaries. In the *rad27*Δ plot these include sections 1, 0, and 2, at least close to the fountain origin (de novo insertions with and without TMH and direct duplications, respectively; Fig. 4f). In other areas, rates exhibit discontinuities at or near class boundaries. These include sections 3 and 4 in the *rad27*Δ plot (i.e. insertions, either with TMH or in repeat tracts, respectively). Note that this discontinuity is not driven by a lack of target sequences (Fig. 4b), as illustrated by detection limits which transition smoothly across the class boundary (Fig. 4c). The boundary is sufficiently noisy to make it unclear whether the trough of the discontinuity lies on the class boundary or just inside the lower edge of section 4. This implies that insertions with TMH are created by a different mechanism than are most insertions in repeat tracts.

While suggestive, discontinuities and continua are insufficient to prove differing or shared mechanisms, respectively, in the absence of plausible rationales. For instance, a mechanism that creates class 0 de novo insertions (no TMH) by inserting novel sequence into random loci also would be expected to also create class 1 insertions with minimal TMH due to random chance. The probability of the first inserted base pair matching the first base pair of a random locus should not depend on insertion length. Therefore, the ratio of class 0 to class 1 de novo insertions should not vary with insertion length. In the *rad27*Δ strain, for de novo insertions of lengths 1, 2, 3, or 4+ base pairs, insertions with no TMH outnumber those with one base pair of TMH roughly 4-to-1 (28:6, 9:2, 8:2, and 8:2, respectively). Insertions with 1 bp of TMH outnumber those with 2 bp of TMH by 6-to-1 (12:2), an insignificant difference at those low counts. This suggests that the same mechanism is responsible for 67 insertions for which the following statement is true: 0 < HL ≤ 2 ≤ ML. Above 2 bp of TMH, class 1 and 2 insertions are too numerous and for the same logic to hold and another mechanism is required.

### The effects of *RAD27* deletion on point mutation rates are mildly affected by ploidy

Ploidy can affect mutation rates^42,97^. Overall mutations rates in the *rad27*Δ diploid were 1.7x lower than in the haploids, a small but highly significant difference (Supplementary Fig. 4; *p* < 2 × 10^−10^; still higher than found in a previous study^87^). Most of the decrease (82%) appears to be attributable to a 2.3× decrease in multi-base insertion rates. However, closer inspection shows a consistent effect on most haploid and diploid indel rates, comparing mutations binned by mutation length and local homology length (Supplementary Fig. 6c, R^2^ = 0.98) or by mutation type (Supplementary Fig. 6d, R^2^ = 0.89). Discussion will focus on the former as it is the more robust data set (187 shared fountain plot bins vs. 14 shared fountain plot sectors with 1 bp indels treated separately from sectors 4 and 7). These are log-log plots in base ten, so a linear correlation with a slope near unity implies a uniform effect on the underlying mutational processes. A y-intercept of 0.12 means a 1.3× difference (i.e. 10^0.12^), also within error of unity. Though most shared bins are due to multi-base insertions, a uniform, mutation-type-independent effect is supported by the fact that none of the 32 deletion, substitution, 1 bp insertion, or complex mutation bins emerge as obvious outliers.

The 1.7× lower overall mutation rate in the *rad27*Δ diploid could be explained by a hypothetical misestimation of elapsed generation counts. However, a 1.7× change in cell divisions would mean only 17.6 generations per passage rather than 30, causing massive decrease in colony volume (2^30-17.3^ = 5200×, depending on growth assumptions). Large differences in generation counts are precluded by selection of colonies of predetermined size (see Methods). Strong selection could theoretically break the correlation between colony size and elapsed generations, but there is no obvious evidence of such selection. In fact, the slightly faster growing diploids, where selection should be weaker, had lower mutation rates.

### *RAD27* deletion increases loss of heterozygosity

Some loss of heterozygosity (LOH), wherein a heterozygous locus becomes homozygous, was already detectable in the *rad27*Δ haploids via combined CNVs and point mutations. Three point mutations occurred in CNV loci during the experiment, followed by LOH later in the experiment. Twelve more occurred in CNV loci before the experiment, followed by LOH during the experiment. 55 point mutations predated the experiment and were then caught in CNVs during the experiment. Given the small fraction of mapped base pairs involved in positive CNVs, this represents a high LOH rate.

214 LOH loci were detected in the *rad27*Δ diploid strain, compared to 727 mutations that were not caused by LOH. This represents a rate of 2.4 LOH loci generated per Gpb per generation. This is 69× the LOH rate of the WT set and about half the rate of the MMR- set. However, simple LOH rates are misleading when diploid strains begin with very little heterozygosity. If heterozygosity is driven by mutations accumulated after isogenic diploidization, then strains with higher mutation rates will have proportionally more heterozygosity and thus more opportunity for detection LOH. Instead, the fraction of variants attributable to LOH (LOH fraction), accounts for differences in heterozygous site density at the cost of measuring absolute rates. The diploid *rad27*Δ LOH fraction was 21.7%, almost twice that of WT and MMR- sets (12.7 and 11.8%, respectively).

## Discussion

**Fig. 5:**
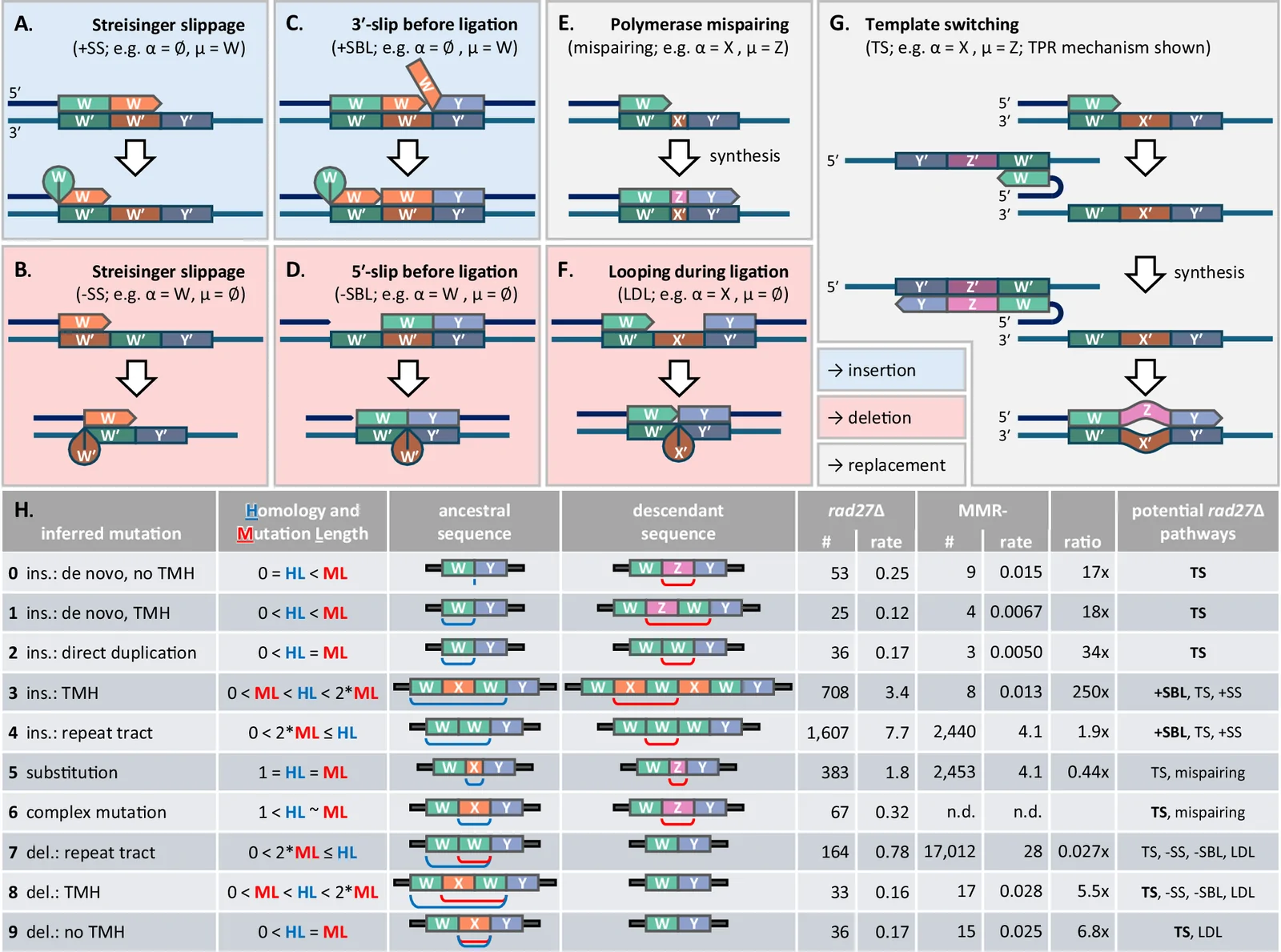
Mechanisms implied by mutation length and sequence context. **A**–**G** Diagrams of mutagenic pathways during DNA synthesis and/or ligation. Mechanisms that cause insertions (∅ → W or ∅ → XW), deletions (W → ∅ or XW → ∅), or segmental replacements (X → Z) are shown on blue, pink, and gray backgrounds, respectively. For simplicity, only the pathway steps that distinguish the mechanisms are illustrated. Notes: α, reference sequence; µ, mutant sequence; ∅, the absence of base pairs; TMH, terminal microhomology. **A**, **B** Streisinger slippage (SS) occurs when repeat tracts or sequences with TMH misalign during replication, generating primer-strand loops (+SS insertions) or template-strand loops (-SS deletions). **C**, **D** Slippage before ligation (SBL) may occur after synthesis but before nick sealing. Template-strand slippage can permit flap complementation and generate insertions (**C** +SBL; see Supplementary Fig. 6a, b for upstream slippage). Primer-strand slippage can generate deletions (**D** −SBL; 3′-slippage in Supplementary Fig. 6c). **E** Replicative mispairing results when a polymerase incorporates an incorrect nucleotide; extension from the mismatch produces a substitution in the next replication cycle. Multiple nearby errors can generate complex mutations. **F** If 5′- and 3′-ends can be ligated across a gap, the resulting template looping during ligation (LDL) could cause a deletion. **G** Template switching (TS) occurs when a dissociated 3′ primer terminus transiently primes synthesis at an alternative template before returning to the original template. During replication, this process is termed transient primer relocation (TPR), a large-scale form of transiently initiated mutagenesis (TIM^86^). After replication, homologous recombination (HR) repairs strand breaks via a highly regulated form of primer relocation involving strand invasion (reviewed^113^). Error-prone homoeologous recombination results when Rad51 filaments invade similar but non-identical DNA sequences. Because homologous flanks are not included in mutation calls, they contribute to neither local homology length (HL) nor mutation length (ML). Depending on the size and sequence of the copied segment, TS can generate substitutions, complex mutations, insertions, deletions, and some copy-number variants (CNVs). **H** Mutation categories organized by ML and HL, showing counts, rates (Gbp⁻¹ gen.⁻¹), *rad27*Δ/MMR- rate ratios, and candidate mutational pathways. Favored pathways are indicated in bold. Source data are provided as a Source Data file.

As mentioned above, elevated *rad27*∆ rates could conceivably be explained by three nonexclusive mutagenic routes: 1) loss of MMR efficiency; 2) loss of damage repair efficiency leading to unrepaired lesions or increased error-prone translesion synthesis; and/or 3) aberrant processing of DNA flaps. Each route is potentially achievable through multiple mechanisms, so we also assess support for each mechanism. We consider seven models for point mutagenesis during genome replication and repair (Fig. 5a–g): forward and backward Streisinger slippage; 3′- and 5′-terminal slippage before ligation; polymerase mispairing; looping during ligation (LDL); and template switching (TS). We relate these models to Rad27p functions (Fig. 1) and the mutational modes discussed above. We also discuss which *rad27*Δ mutations are best explained by each model, the drawbacks of the mutation accumulation as a method for inferring mechanism, alternative models, and future experimental opportunities.

### MMR and RER defects cannot explain *rad27*Δ indels in repeat tracts

There is functional redundancy between the Exo1, Rad27, and Mlh1-Pms1 excision pathways during MMR^21,98^ (Fig. 1e, f). *RAD27* deletion could therefore only cause a partial MMR defect at best. We checked each mutation type for evidence of such defects.

Streisinger slippage (SS) occurs when replicating repeat tracts or tracts with terminal microhomology^99^, causing local primer or template strand extra-helical bases. If not repaired before the next replication round, these respectively result in insertions (+SS; Fig. 5a) and deletions (−SS; Fig. 5b). These are the most frequent errors for replicative polymerases, resulting in the dominance of class 4 insertions and especially class 7 deletions in MMR- strains (Fig. 5h). As such, MMR defects caused by a lack of *RAD27* would be expected to cause class 4 and 7 indels in similar proportions. This is not the case in *rad27*Δ cells, where class 4 insertion rates exceed those of MMR- cells. An MMR defect in repairing -SS events cannot be fully excluded as an explanation of class 7 deletions. However, *rad27*Δ class 4 insertion rates cannot be explained by an MMR defect and are thus unlikely to be due to +SS events during replication.

The comparison between wild type and MMR- strains suggests that MMR more efficiently repairs extra template strand As or Ts more efficiently than extra template strand Gs or Cs. This difference could be related to the fact that a G•C base pair has one more hydrogen bond than an A•T base pair, such that more energy is required to create an unpaired G or C than an unpaired A or T^89^. The lesser A versus G deletion imbalance in the *rad27*∆ strain means that either Rad27p participates specifically in MMR of G deletions or that elevated *rad27*∆ rates are not entirely due to an MMR defect.

In the *rad27*∆ cells, the single base addition rate is 4-fold *higher* than the single base deletion rate. This differs from the MMR- strains, wherein the single base addition rate is 8-fold *lower* than the deletion rate (Fig. 2a). We did not anticipate this difference ab initio because the nuclear replicases make single-base deletions 10-fold more often than single base additions during DNA synthesis in vitro (reviewed^89^). Such a preference would make sense because generating an unpaired primer strand loop via SS requires disrupting at least one extra base pair than does an equivalent template loop, making deletions energetically favored^89^. However, in addition to the insertion bias in the *rad27*∆ spectrum, its single-base deletion rate is also 3% of that in MMR- strains (Fig. 2C). This is consistent with roles for other nucleases in patch/flap removal during MMR.

It is still technically possible that a minority of these insertions could result from loss of Rad27-dependent MMR via the MutSβ (Msh2•Msh3) pathway, which is specialized for 2–15 bp indel loops recognition. However, a large number of indels in the *rad27*∆ strain, and not in the MMR- strains, exceed this size (Fig. 4e, f). This lends further credence to the hypothesis that many *rad27*∆ indels are not driven by an MMR defect.

Defects in ribonucleotide excision repair (RER) cause characteristic deletions in direct repeat tracts with repeat units of 2–5 bp (reviewed^100^), with nearly all dinucleotide deletions in RER-deficient yeast found in in AC or AG repeats^94^. There is evidence for Rad27p involvement in the resolution steps of this repair pathway (Fig. 1k^23,70^;). However, over three quarters of deletions in *rad27*∆ cells fall outside of the 2–5 bp range and few were in AC or AG repeats. Therefore, a potential RER defect is excluded as a major contributor to the *rad27*∆ mutation spectrum.

### Slippage before ligation at 5′ flaps likely causes repeat and TMH insertions in *rad27*∆ cells

Biochemical studies show that Fen1 acts on dynamic, equilibrating flap intermediates, including double-flap substrates, supporting the idea that unprocessed flaps can rearrange prior to ligation^16,101–103^. After replication, it is possible for either end of a primer strand to slip before ligation (SBL). Such slips can make room for re-equilibration of a 5′-flap (Fig. 5c, Supplementary Fig. 6a), or additional replication (analogous to +SS; Supplementary Fig. 6b), causing an insertion in the next replication round (+SBL;). Alternatively, if slippage closes a gap then a deletion will occur (−SBL; Fig. 5d, Supplementary Fig. 6c). In either case, the 5′-end may either precisely encompass a repeat unit, causing repeat indels (classes 4 and 7), or may bridge non-homologous DNA, causing indels with TMH (classes 3 and 8). Class 3, 4, and 8 indels are all elevated in the *rad27*∆ strain relative to the MMR- strains, meaning that they cannot be attributed to an MMR defect alone.

Two of these mechanisms, both of which would cause insertions (+SBL), involve 5′ flaps and thus are likely to result from *RAD27* deletion. Thus, we suggest that slippage and re-equilibration of unrepaired 5′ Okazaki flaps caused nearly all of the 708 repeat insertions (class 3) and most of the 1607 insertions with TMH (class 4) in the *rad27*∆ strain (Fig. 5h). A + SBL mechanism, similar to the one illustrated in Supplementary Fig. 4a, was previously proposed for insertions in *rad27*∆ yeast^87^. We suggest that the version illustrated in Fig. 5c is equally likely, as it would disrupt the same number of hydrogen bonds. Mutations explainable via +SBL are the most common mutation types in the absence of *RAD27*.

If these indels, primarily multi-base insertions, are indeed caused by defects in OFM, then they should be concentrated where OFM is most common, for example at replication origins. We previously used ribonucleotide incorporation during nuclear DNA replication^27^ to test a proposal by O’Donnell and colleagues^104^ that replication of both the leading and lagging DNA strands is initiated at replication origins by synthesis of an Okazaki fragment. When mapped against the whole genome, multi-base insertion rates peak on either side of replication origins (Fig. 3e). The centroids of these peaks are, on average, just on the origin side of the +1 and −1 nucleosomes, as was previously observed for Rad27p-cleaved OF termini^105^. This strongly suggests that some of the *rad27*∆ multi-base insertions are associated with OF termini.

### Replicative mispairing may cause substitutions in *rad27*∆ cells

Replicative mispairing (Fig. 5e) occurs when a polymerase mistakenly mispairs an incorrect incoming base with the template. Extension from this mispaired terminus will cause a substitution mutation in the next replication round. This is technically the smallest form of segmental replacement. Clustered or consecutive mispairs or combinations of mispairs and slippage during a single synthesis event would cause a longer complex mutation. Such error-prone synthesis is rare for replicative polymerases but common for less accurate polymerases like Pol ζ^106^. Substitution (class 5) rates are only somewhat lower in the *rad27*∆ strain than in the MMR- strains (56% lower; Fig. 5h). Compare this with repeat deletions (class 7), where *rad27*∆ rates are 37-fold lower than MMR- rates (Fig. 5h). Substitutions are normally repaired via the MutSα sub-pathway of MMR while repeat deletions are repaired via both MutSα and MutSβ. This difference in ratios could indicate a MutSα-specific MMR defect in *rad27*∆ cells. However, the MutSα sub-pathway is also the primary pathway for repair of 1 bp deletions, and those are also much lower in *rad27*∆ cells (34-fold; Fig. 2a). It is difficult to rationalize a decrease in MMR of substitutions via a Rad27p driven defect in the MutSα pathway that would not have a larger effect on 1 bp deletion rates.

If loss of MMR efficiency causes elevated *rad27*∆ substitution rates, then it is no more than a partial loss, with *rad27*∆ substitution rates being 45% of the rates in MMR- cells (Fig. 2C). A partial loss of MMR in the *rad27*∆ strain would be consistent with the participation of alternative nucleases in MMR, potentially including exonuclease 1 (Exo1)^21^, the proofreading 3′-exonucleases DNA Polymerase (Pol) δ and ε^107^, MutLα^24^, exonuclease Dna2. The latter has also been implicated in both long-patch Okazaki fragment maturation (OFM; Fig. 1e; reviewed^47^) and in MMR^108^.

The 4.7-fold higher rates of G•C to C•G mutations in the *rad27*∆ strain (Fig. 2C, blue) suggests that some of the responsible mismatches may contain damaged bases in G•G or C•C mismatches that use Rad27p for removal during BER, NER or some other process. The most likely error-prone damage tolerance pathways involve translesion synthesis. The particularly relevant error prone translesion polymerases in *S. cerevisiae* are Rev1, Pol η, and Pol ζ. Rev1 is a poor candidate, as it preferentially by incorporating C across from lesions and abasic sites (^109^ and references therein), generating substitutions to C or G, whereas changes to A or T dominate substitutions in the *rad27*Δ strain. Pol ζ has a high propensity for G•G mispairing in vitro^110^ and elevates G•C to C•G and A•T to T•A transversions when it appears to assist bulk replication^111^. These are the two most elevated substitutions in *rad27*Δ strains, relative to MMR- strains (Fig. 2c), but their rates are still less than those of G•C to either A•T or T•A. In summary, the *rad27*Δ base substitution spectrum is clearly consistent with a significant role in repairing a subset of base•base mismatches made during replication of undamaged nuclear DNA. At the same time, the results suggest that Rad27p has additional roles in modulating nuclear genome stability.

Could the complex mutations in the *rad27*Δ strain reflect Pol ζ activity secondary to repair defects (BER, NER, etc.)? After all, Rad27p may be involved in NER^74,75^ (Fig. 1m) and Pol ζ generates complex mutations in vitro and elevates complex mutation rates in NER-defective cells^106,110,112^. In some studies, a mutator variant of Pol ζ was used to accentuate this phenotype. These Pol ζ-dependent mutations were not explained by conversions or hairpin/pseudo-palindrome extension and predominantly involved GC or TC bases (79%) converted to AA or TT (72%) in vivo. Six of eleven tandem substitutions in the *rad27*Δ strain created CC dinucleotides, and none originated from GC or TC dinucleotides. All could be attributed to either conversions or hairpin/pseudo-palindrome extension. Thus, the complex mutation spectrum in *rad27*Δ appears to differ from that associated with Pol ζ.

### Looping during ligation is unlikely to contribute to deletions in *rad27*∆ cells

If 5′- and 3′-ends can be ligated across a gap, the resulting template looping during ligation (LDL) could cause a deletion (Fig. 5f). This mechanism does not require flanking sequence homology and thus could result in deletions of classes 7, 8, and 9 (Fig. 5h). It is unknown whether such ligation across a gap is possible with the ligases present in *S. cerevisiae* nuclei. It is also difficult to see why ligation across a gap would be more common in *rad27*∆ cells than in MMR- cells. Thus, while we include LDL in the discussion as a technical possibility, we do not consider it a good candidate explanation for *rad27*∆ mutagenesis.

### Template switching is consistent with many *rad27*∆ mutations

A 3′-primer terminus that dissociates from its template can relocate to an alternative template (Fig. 5g). Following some amount of synthesis, a second relocation event could return the new nascent end to original template. Such template switching (TS) could cause segmental replacements either during (S phase) or outside of normal replication (G2 or late S phase).

During replication, transient primer relocation (TPR) is an extreme form of transiently initiated mutagenesis (TIM^86^). TPR requires the relocation of a 3′-primer terminus, but 5′-flaps are the structure on which Fen1 most famously works. Why would unpaired 3′-flaps arise? The ideal substrate for yeast Fen1 is actually a dually flapped substrate^102^. This could be created via partial re-equilibration of 5′-flap, which would disrupt base pairs on the opposite terminus. While yeast Fen1 prefers a substrate with a 1 bp 3′-flap and a longer 5′-flap, runaway re-equilibration could create extensive 3′-flaps in the absence of proper 5′-flap processing.

Homologous recombination (HR) involves the coordinated action of many proteins for strand invasion and resolution (reviewed^113^). Homoeologous recombination is an error-prone mode of HR involving similar but not identical DNA sequences. HR most commonly occurs during either meiotic crossover or during double strand break repair (DSBR). Constant nutrient abundance during mutation accumulation experiments encourages vegetative reproduction over meiosis and its associated recombination. The most likely candidate would be homoeologous recombination due to erroneous synthesis-dependent strand annealing (SDSA), (reviewed^113^). SDSA could be thought of as TPR with more steps, such as Rad51 nucleoprotein filament formation (requiring Rad51 and/or Dmc1), strand invasion, and junction resolution. Notably, HR is absolutely required for viable haploid cells in the absence of Rad27 activity, which was attributed to repair of frequent double-stranded breaks^114,115^. Previous substitution spectra from *rad27*∆ cells also have high cosine similarity to spectra from cells with individual deletions of several HR components including *RAD51*^116^.

Depending on relative size and sequence of the ancestral and descendent blocks between these flanks, TS can cause any of the nine mutation classes. These include substitutions, complex mutations, and indels from as small as one base pair to those large enough to constitute copy number variants (CNVs). The *rad27*∆ CNV rate is 4.9-fold higher than the rate in *msh2*Δ cells and CNV types differ. This again precludes attribution of *RAD27* knockout effects to an MMR defect alone. In another contrast with *msh2*Δ cells, many *rad27*∆ CNVs encompass chromosome ends. Non-transient primer relocation events could easily explain these CNVs.

TS is the only mechanism that explains the de novo insertions (classes 0 and 1) and direct duplications (class 2) observed at high rates in *rad27*∆ cells (Fig. 5h). TS is also the only mechanism that explains the 57 of 67 *rad27*Δ complex mutations that were found to be segmental replacements, including 30 that required switching chromosomal templates (Fig. 2e). For class 9 deletions (no TMH), the only two explanations are TS or LDL, with TS by far the preferred explanation (see above for objections to LDL).

### Assigning mechanisms to *rad27*∆ mutations

Insertions due to strand slippage before ligation (+SBL) are likely the main source of mutations in the *rad27*∆ strain. Class 3 and 4 insertions (in repeat tracts or with TMH) could be explained by TS. However, because TS can explain any of the nine mutation classes, caution is warranted. TS should only be invoked when the sequence of the mutation and its flanking context are present elsewhere in the genome and evidence and/or logic also weigh against all possible alternatives. For these insertions, +SBL is both the simpler mechanism and it comports with the known functions of Rad27p. Therefore, we assign the bulk of class 3 and 4 insertions to +SBL. With the wild type background being negligible (Fig. 2a), this suggests that +SBL causes around 61% of *rad27*∆ mutations.

Conservatively, at least 12% of the observed *rad27*∆ mutations must be due to TS (397 of 3285 point mutations and CNVs). This represents all mutations of classes 0, 1, 2, and 9, plus 82% of class 8 (deletions with TMH; allowing for a maximal MMR defect), plus the 57 complexes resulting from segmental replacement, plus 95% of non-aneuploidy CNVs (allowing for the wild type background rate).

The actual fraction of *rad27*∆ mutations due to TS is likely much higher. We discussed above why replicative mispairing revealed by an MMR defect is unlikely to explain all substitutions and why we see little evidence of substitutions due to repair polymerases. Similar logic applies to -SS for class 7 deletions (repeat tracts), which could theoretically be due to TS, -SS, -SBL, and/or LDL. There is no obvious reason why -SBL would be more frequent in the absence of Rad27p, even given all of the processes in which Rad27p is involved, so we will set -SBL aside for now. This leaves only TS to explain up to 89% of the 383 substitutions and nearly all 164 class 7 deletions (accounting for the wild type background in Fig. 2a). These raise the total estimate to 27% of *rad27*∆ mutations caused by TS, or four mutations per Gbp per generation. How much higher would the TS rate be if *RAD27* was deleted in combination with key components of alternative flap removal pathways, such as Exo1 or MutSα^22–24^? How many TS events are invisible to mutation accumulation experiments because the primer relocates to a truly homologous locus? Note that the point mutation rate in the diploid *rad27*Δ strain is lower than in the haploid (Supplementary Fig. 4a) but the LOH fraction is higher (Supplementary Fig. 4e). This implies that increasing chromosome copy number increases accuracy, possibly by providing more opportunities for more homologous (invisible) or nearly homologous TS (LOH).

More work is required to distinguish between alternative TS mechanisms. In the meantime, we provisionally favor replication-associated TPR as a major contributor to TS events in *rad27*Δ cells. Notably, these cells still retain active MMR components such as MutSα, which suppress recombination between diverged sequences (homoeologous recombination; reviewed^113^). Diploid *rad27*Δ cells have lower mutation rates (Supplementary Fig. 4a, c), yet show a higher fraction of loss-of-heterozygosity (LOH) events relative to MMR-deficient strains (Supplementary Fig. 4e). This suggests rampant *rad27*Δ TS events even when homoeologous recombination should be repressed. Distinguishing between replication- and recombination-associated TS will require future experiments, for example by defining the timing of TS events during S phase.

The complicated mutation spectrum of the *rad27*∆ strain is unique compared to other defective yeast strain we have previously examined. The results indicate that loss of Rad7p increases rates for mutations resulting from defects in OFM, with lesser contributions from defects in DNA mismatch repair, ribonucleotide excision repair, and perhaps other types of DNA repair. The present study poses many new issues that can be addressed in the future. For example, this study investigates the consequences of complete deletion of the *RAD27* gene but leaves open the issue of defining the roles of each of the three activities of Rad27/Fen1 (Fig. 1a–c) in modulating these consequences. This could be investigated in the future using *RAD27* mutants that alter each of these activities individually. In a similar fashion, mutation specificity can be investigated using *rad27*Δ mutant strains harboring additional mutations in other genes. Given our own interest in DNA replication fidelity, we have already introduced mutator mutations in each of the nuclear replicases into the *rad27*∆ strain, to investigate their potential roles in Rad27-dependent MMR, OFM or RER (reviewed^117^). For example, similar experiments could be initiated for genes that encode proteins operating in any of the other DNA transactions in which Rad27p has been implicated. Additionally, an explanation is needed for the decrease in insertion rates in late replicating *rad27*∆ cells (Fig. 3i). *RAD27* deletion increases dNTP pools via interactions between Rnr1, Dun1, and Sml1^118^. Such pool increases should favor extension from erroneous structures and thus increase error rates. More work is needed to resolve this contradiction and to determine whether *rad27*Δ-induced replication stress alters origin usage and replication timing.

It should also be noted that mutation spectra in one organism are only one line of evidence required for proving a mutation mechanism. In vitro error profiles of purified components and structures with Rad27 bound to mutational intermediates would be supportive. Finally, because rates for so many types of mutations are elevated by loss of Rad27p in yeast, it will be of great interest to examine whether mutations in the human Rad27 gene are associated with development of human diseases.

## Methods

### Strain construction and naming

The *Saccharomyces cerevisiae* strains used here^33,85,96^ were isogenic diploids derived from the S288c, W303, or Δ7 backgrounds. The latter is also known as Δ|(−2) | −7B-YUNI300 (*MATa CAN1 his7-2 leu2*Δ::*kanMX ura3*Δ *trp1-289 ade2-1 lys2ΔGG2899-2900*)^119,120^. Strain names used here are simplified from the standard genotype nomenclature. For example, the standard haploid *rad27*Δ*::HYG* simplifies to *rad27*Δ and the diploid *msh2*Δ*::HYG/msh2*Δ*::HYG* becomes simply *msh2*Δ. Full genotype descriptions may be found in the referenced studies.

Strains were created as previously described (Table 1)^121^. Briefly, isogenic diploids were generated from haploid cells via transformation of the mating type switching plasmid YEpHO^122^. Diploid clones were selected. One copy of the target gene (e.g. *RAD27*) was deleted and replaced via transformation with a PCR product containing the HYG-resistance cassette and 300 nucleotide flanks homologous the regions upstream and downstream of the target ORF. Transformants were selected for, and target gene deletion and replacement were verified by both PCR and phenotype. The resulting diploids were sporulated and spores were selected for HYG resistance. Homozygous diploids were created by back-crossing with Mat a and Mat α samples from spore colonies. Diploids were selected for inability to mate to Mat a and Mat α haploids.

Strains other than *rad27*Δ were collected into strain sets based on the presence (WT) or absence (MMR-) of DNA mismatch repair (Table 1). The WT set is composed of Δ7^33^, W303^26,111^ and S288c diploid yeast. The MMR- set is composed of *msh2*Δ^85^, *mlh1*Δ^84^, and *pms1-DE*^96^ strains in the Δ7 background and an *msh2*Δ strain in the S288C background (reported here). Strains within sets have statistically indistinguishable mutation rates, spectra, biases, and distributions (see previous methods^85,96^).

### Mutation accumulation and genome sequencing

Whole genome mutation accumulation experiments were conducted as previously described^33,85,96,123^. Briefly, independent isolates of each strain were passaged on solid media (YPDA + 100 µg/mL adenine) with single-cell bottlenecks when the median colony grew to ~2.5 mm in diameter (2.5–4 days at 30 °C, depending on strain), roughly every thirty generations. Haploid cells lacking RAD27 grow about 50% slower than wild type^124^, requiring an additional day of growth per passage, on average, and seven of fifty isolates failed to propagate. Growing colonies to the same size ensures a similar number of elapsed cellular generations, assuming similar cell volumes and rates of cell death. These bottlenecks fix mutations in the population and discourage competition and differential selection due to local nutrient depletion. Samples were collected at the beginning of the experiment (time zero samples), and periodically throughout the experiment. If, before the last intended passage, a prohibitive growth defect was acquired, then the sample from the last pre-defect passage was used instead. No prohibitive growth defects were encountered with *rad27*Δ cells. Please see cited publications (Table 1) for details of other strains.

Mutation rates from existing reporter gene assays suggested ~20,000 generations were needed to obtain at least 100 examples of each major mutation type in *rad27*Δ haploid cells. Fifty isolates and 15 passages were selected to give 10% padding. Reporter gene estimates proved to be low, so the lowest mutation count by type (>1 bp deletions) still passed the threshold despite the seven isolates that failed to propagate. For *rad27*Δ diploids, it was decided that only 20 mutations per type were needed for statistical comparison to haploids. 22 outgrowths for 6 passages were predicted to be more than sufficient. Diploid rates were 70% lower than expected but still yielded at least 25 mutations per type (again >1 bp deletions). Sample counts for wt and MMR- strain sets were fixed by what had already been published or was on hand from other studies.

DNA was extracted, sequencing libraries were prepared, and their genomes sequenced as per the original publications (Illumina Inc.; 2 × 150 bp paired end reads on; platforms varied with year of collection; see Sequence Read Archive BioProject PRJNA245050 for platforms by sample)^33,85,96^. Software used in sequencing data collection: Illumina platform on-board software bcl-convert 4.2.4; fastq_screen v0.6.4; FastQC 0.12.1; and iRODS & NextFlow, version unknown.

### Mutation calling

The *muver* pipeline was used to call and classify variants that accumulated during the experiment (version 1.2.4; dependencies: samtools 1.18; picard tools 2.27.5; bowtie2 2.5.2; GATK 3.8-1-0-gf15c1c3ef; Python 3.12.3; and NumPy 1.26.4)^83^. Briefly, after reads were mapped to the reference sequence (S288C, W303, or version 2 of the *S. cerevisiae* L03 reference^85,123^), *muver* found loci with depths that differed significantly from the sample median. These were excluded from analysis by default but were also manually assessed to detect copy number variation (CNV). CNV loci were reported to *muver* for inclusion with adjusted local ploidy and variants were then called. *Muver* detected initial- versus final-sample allelic fraction differences and applied a series of statistical filters and genotype models to determine which variants could be classified as point mutations. A quality control step evaluated samples for potential cross-contamination, ploidy changes, sample switching, or forked lineages (for contamination and ploidy detection, see ref. ^83^). No issues were discovered for wild type W303, wild type S288C, or S288C *msh2*Δ samples. Three forked lineages were detected in *rad27*Δ samples (pairs TAK2569/2570, TAK2591/2592, TAK2593/2594). Duplicate mutations were removed from one sample in each pair (TAK2569, 2591, and 2593, respectively) and the number of elapsed generations discounted according to the fraction of mutations removed (set to 170, 222, and 150 generations, respectively). Please see cited publications (Table 1) for details of other strains. A sample was excluded if sequencing failed entirely, or if depth was insufficient (<30x; 2 of 179 outgrowths), or if its mutation rate was outside of the 99.9% confidence envelope by strain (5 of 179 outgrowths; all high, implying acquisition of cryptic mutator mutations).

Mutation rates were calculated as previously reported^33^. See Table 1 and Fig. 2a for generation counts, queryable base counts, and mutation counts by strain and by strain set. See Supplemental File 1 for annotated variant lists. Complex mutation candidates were defined as loci wherein multiple individually called mutations in one sample were discovered with separation of under 12 bp. The sequencing data were assessed manually and algorithmically to ensure that the component mutations were properly phased (found on the same sequencing read, and therefore on the same chromosome). Unfortunately, as mutation density increases in each outgrowth line, so does the probability that two independent mutations will occur close together. If the goal is to call mutation *events* with minimal false positives, then high mutation density precludes confident complex mutation calling. Complex mutations and their flanking sequences were compared to the reference genome via BLAST^125^. Complex mutations were assigned as hairpin or pseudo-palindrome extensions of the mutation increased the length of a hairpin or pseudo-palindrome within 30 bp of the mutation loci. Complex mutations were assigned as segmental replacements if a sequence identical to both the mutant sequence and flanking sequence(s) was found elsewhere in the genome and the probability of finding such a good match was under 5%. In practice, approximately 12 matching nucleotides were required to establish statistical significance. Thus, shorter complexes require longer matching flanks to establish significance. If the best match was on the same chromosome, then the complex was assigned as an inter-chromosomal conversion (ICC). If all matching sequences were on other chromosomes, then the replacement was assigned as an extra-chromosomal conversion (ECC). If equally good matches were found on the same *and* other chromosomes, the replacement was called ambiguous. If no good matches were found, the complex was tagged as “unknown”.

### Rate calculations

Mutation rates were calculated as previously reported^83^. Briefly, the rate for mutation type m in isolate j is calculated as

1 $${\mu }_{{mj}}=\frac{{N}_{{mj}}}{{g}_{j}{L}_{{ij}}}$$

where ${N}_{{mj}}$ is the number of mutations of type m in isolate j, ${g}_{j}$ is the total number of generations that separate isolate j from its time 0 ancestor, and ${L}_{{ij}}$ is the number of base pairs in target bin i in isolate j, accounting for ploidy and copy number variation. Overall rates (all mutations, substitutions, insertions and deletions by length, etc.), are averaged across all isolates in the set and the target bin is the whole genome. Rates of specific substitutions (e.g. A•T to G•C) or indels of specific bases (e.g. G•C deletion), the target bin is all bases with the reference identity or its complement (e.g. A + T or G + C, respectively). Rates around features use aggregate mutation counts from all isolates in the set rather than averaging across isolates. With respect to genes, open reading frames, or nucleosomes, each mutation is assigned to the closest gene only, to avoid double counting, and the surrounding bins scale accordingly. Replication time and nucleosome density maps we reported previously^85^.

### Counting genomic targets by length and local homology

Target counting by length and local homology were performed in Python 3.12.3 with the library NumPy version 1.26.4. To calculate the targe size of an insertion or deletion with a given length of microhomology, first all unique subsequences present in the genome were determined by using a sliding window function over each chromosome sequence. Subsequences containing ambiguous base assignments were discarded. Then an index of the start position for all occurrences in each chromosome was recorded.

Repeat tract counts were attained by determining if a given subsequence is comprised of a smaller repeating unit, accounting for repeats of partial length (e.g. CATCATCA with repeating unit CAT). Repeat tracts in telomeric regions were discarded.

Subsequences not comprised of a smaller repeat unit (and not within a larger repeat tract) represent all possible terminal microhomology sites. For each unique subsequence of given length k, the set of indices $\left\{{i}_{1},\,{i}_{2},\ldots {i}_{n}\right\}$ on one chromosome form the basis for a triangular matrix U:

2 $$U=\left[\begin{array}{ccc}{d}_{1,2} & \cdots & {d}_{1,n}\\ \, & \ddots & \vdots \\ \, & \, & {d}_{n-1,n}\end{array}\right]$$

Where d is the distance between two indices. Each distance is a single target for a terminal microhomology mediated indel, with a change in length d and a homology length of either k+d or k for insertions or deletions, respectively. The target size is thus the total count of distances over all chromosomes for all unique subsequences (k = 1–50). The target size for direct duplication events, de novo insertion events, and deletion events with no local homology is the count of all sites of a given length k that are not in a repeat tract.

### Significance testing

Significance was tested using the heteroscedastic Welch’s *t* test^126^ with the degrees of freedom approximated via the Welch-Satterthwaite equation^127^, unless otherwise noted. Significance thresholds were set by applying the Šidák correction for multiple hypothesis testing^128^ for a family-wise error rate of 0.05.

### Reporting summary

Further information on research design is available in the Nature Portfolio Reporting Summary linked to this article.

## Supplementary information

Supplementary Information

Description of Additional Supplementary Files

Supplementary Data 1

Reporting Summary

Transparent Peer Review file

## Source data

Source Data

## Data Availability

Sequencing data has been deposited at the NCBI’s Sequence Read Archive under BioProject PRJNA245050 [https://www.ncbi.nlm.nih.gov/bioproject/245050]. The all mutation calls and the counts and rates derived therefrom in this study are provided in the Supplementary Data files and Source data file. Source data are provided with this paper.
