# Supplementary material for: Two distinct Okazaki fragment failure modes dominate mutagenesis in the absence of flap endonuclease 1
Source: Nat Commun. 2026 Jul 17;17:8749. doi: 10.1038/s41467-026-75267-3 (PMC13493898; doi:10.1038/s41467-026-75267-3)
Supplement: Supplementary file 1 [file 41467_2026_75267_MOESM1_ESM.pdf]

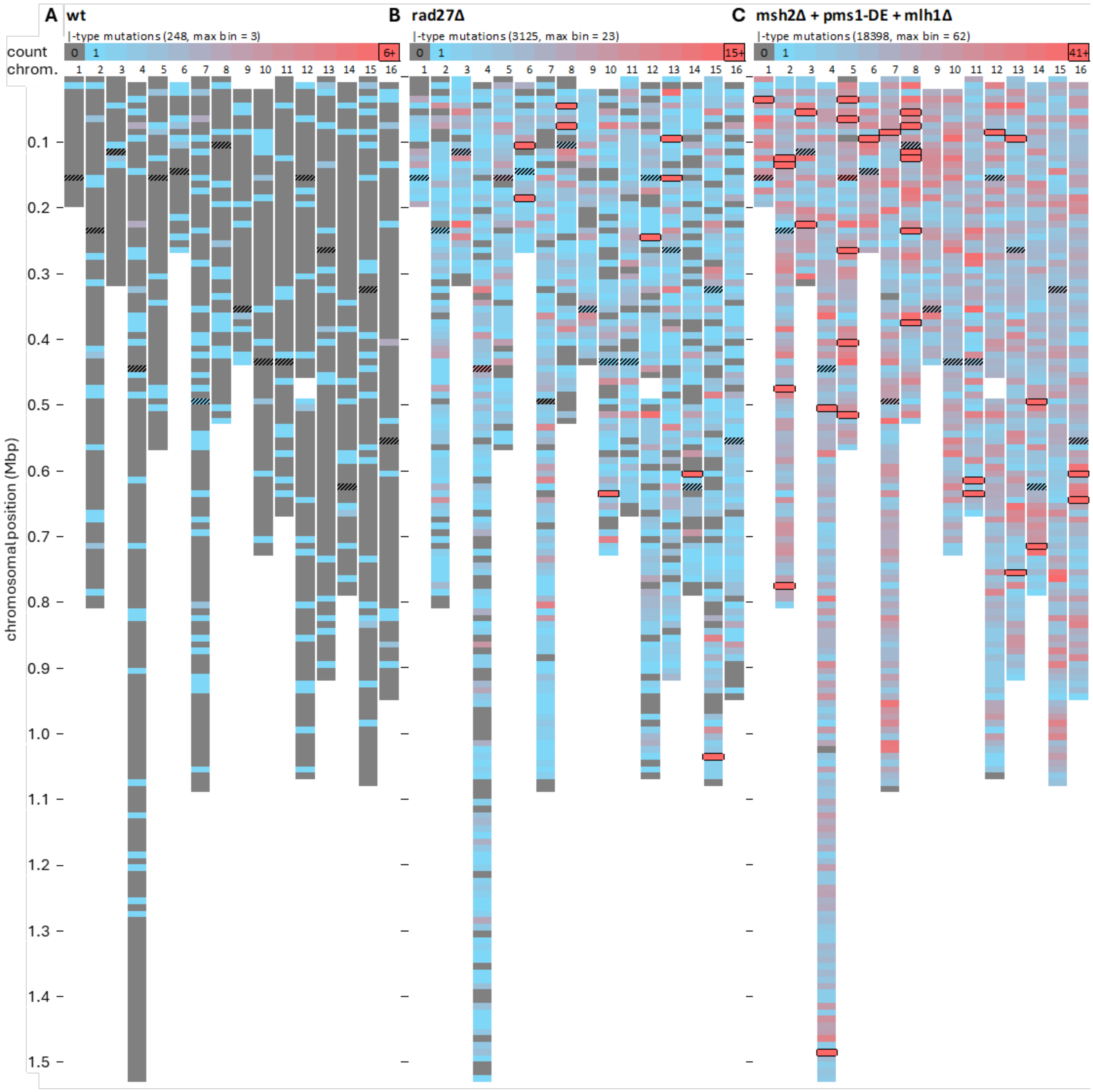

Supplementary Figure 1. Mutation distribution across yeast chromosomes, by genotype. Chromosomal positions of mutations across the 16 yeast chromosomes (Δ7 background): (A) wild type, (B) *rad27*Δ, and (C) MMR- yeast. Bins are colored by mutation density. Bins containing centromeres are hatched. Black-bordered red bins contain more mutations than expected due to random chance, after Šidák correction for multiple hypothesis testing [98] for a family-wise error rate of 0.05.

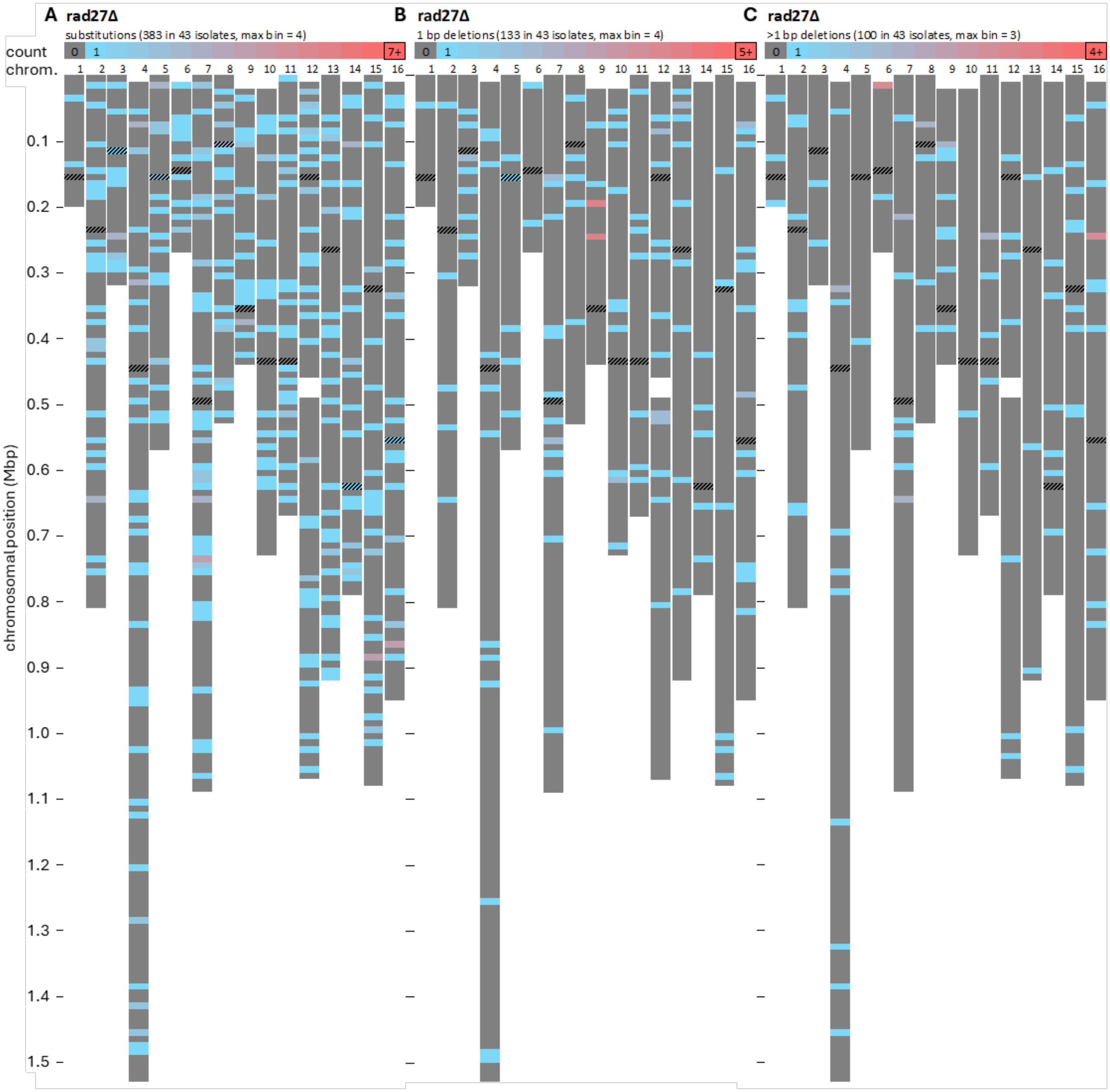

Supplementary Figure 2. Substitution and deletion distribution across *rad27*Δ yeast chromosomes. Chromosomal positions of mutations across the 16 yeast chromosomes in *rad27*Δ yeast: (A) substitutions; (B) single-base deletions; and (C) multi-base deletions. Bins are colored by mutation density. Bins containing centromeres are hatched. No bins contained more mutations than expected due to random chance.

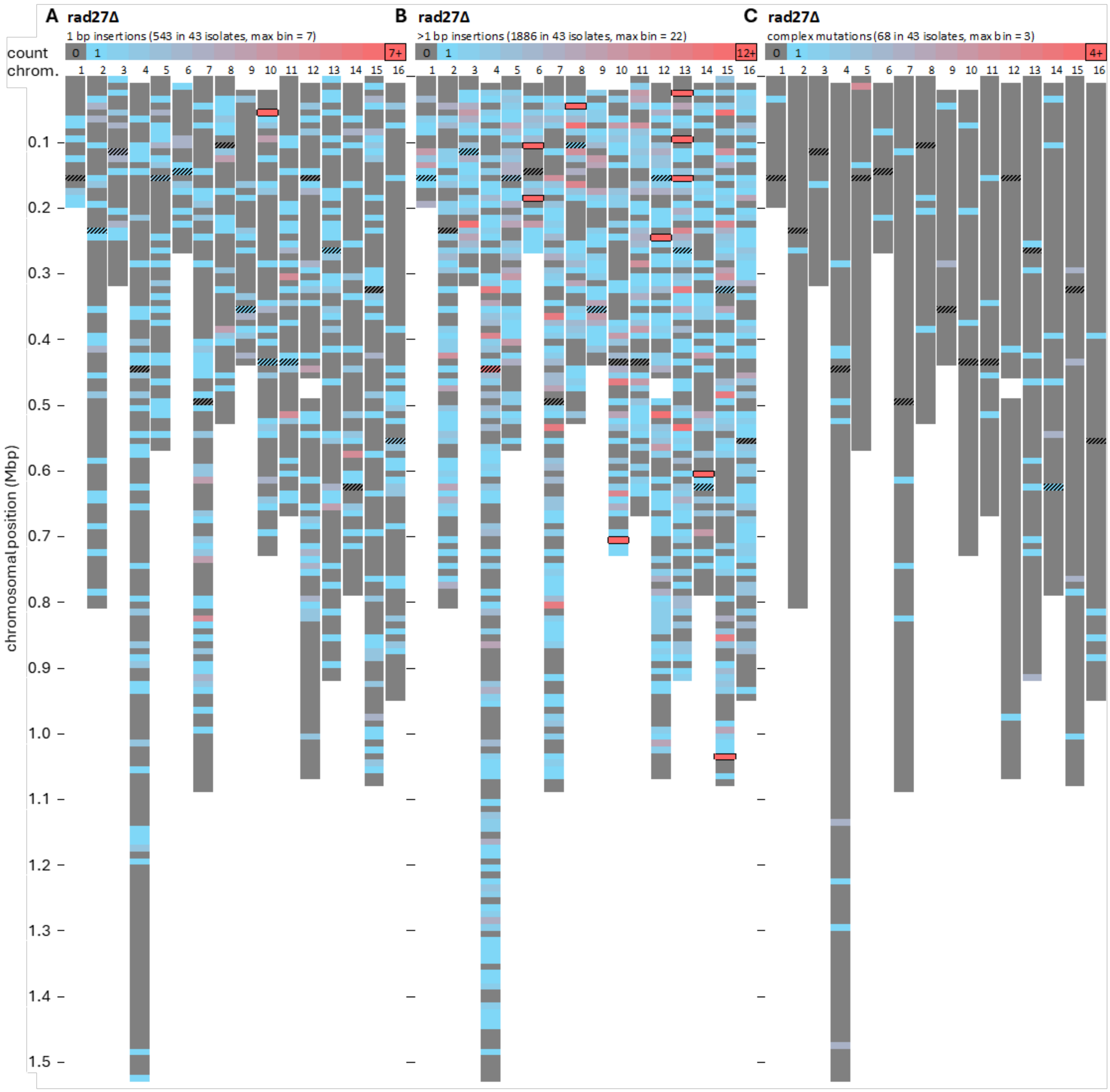

Supplementary Figure 3. Insertion and complex mutations distribution across *rad27*Δ yeast chromosomes. Chromosomal positions of mutations across the 16 yeast chromosomes in *rad27*Δ yeast: (A) single-base insertions; (B) multi-base insertions; and (C) complex mutations. Bins are colored by mutation density. Black-bordered red bins contain more mutations than expected due to random chance, after Šidák correction for multiple hypothesis testing [98] for a family-wise error rate of 0.05.

A	*rad27Δ* haploid			*rad27Δ* diploid		
isolates	43			22		
total gen.	18,524			3,960		
mapped (Gbp)	0.49			0.49		
mutation type	count	rate (Gbp^{-1} gen.$^{-1}$)	rate sterr.	count	rate (Gbp^{-1} gen.$^{-1}$)	rate sterr.
all mutations	3,113	15	0.38	776	8.8	0.46
**substitutions**	383	1.9	0.096	117	1.4	0.13
A|T to G|C	75	0.58	0.057	35	0.64	0.13
G|C to A|T	87	1.1	0.12	23	0.69	0.15
A|T to T|A	84	0.65	0.078	30	0.55	0.11
A|T to C|G	25	0.20	0.038	2	0.037	0.025
G|C to T|A	66	0.83	0.13	21	0.63	0.093
G|C to C|G	46	0.58	0.079	6	0.18	0.064
**1 bp del.**	133	0.64	0.060	36	0.41	0.053
A|T del.	113	0.87	0.088	33	0.61	0.079
G|C del.	20	0.26	0.064	3	0.09	0.049
**> 1 bp del.**	100	0.48	0.066	25	0.29	0.058
2 bp del.	28	0.14	0.038	4	0.046	0.021
3 bp del.	14	0.067	0.02	3	0.034	0.019
4 bp del.	8	0.039	0.012	0	≤ 0.011	
> 4 bp del.	50	0.24	0.10	18	0.21	0.099
**1 bp ins.**	543	2.6	0.13	196	2.3	0.19
A|T ins.	490	3.8	0.20	181	3.3	0.29
G|C ins.	53	0.67	0.087	15	0.45	0.14
**> 1 bp ins.**	1,889	9.1	0.27	353	4.0	0.24
2 bp ins.	705	3.4	0.12	135	1.6	0.18
3 bp ins.	242	1.2	0.089	65	0.74	0.095
4 bp ins.	77	0.37	0.048	13	0.15	0.032
> 4 bp ins.	865	4.2	0.31	140	1.6	0.32
complexes	67	0.32	0.052	49	0.56	0.085

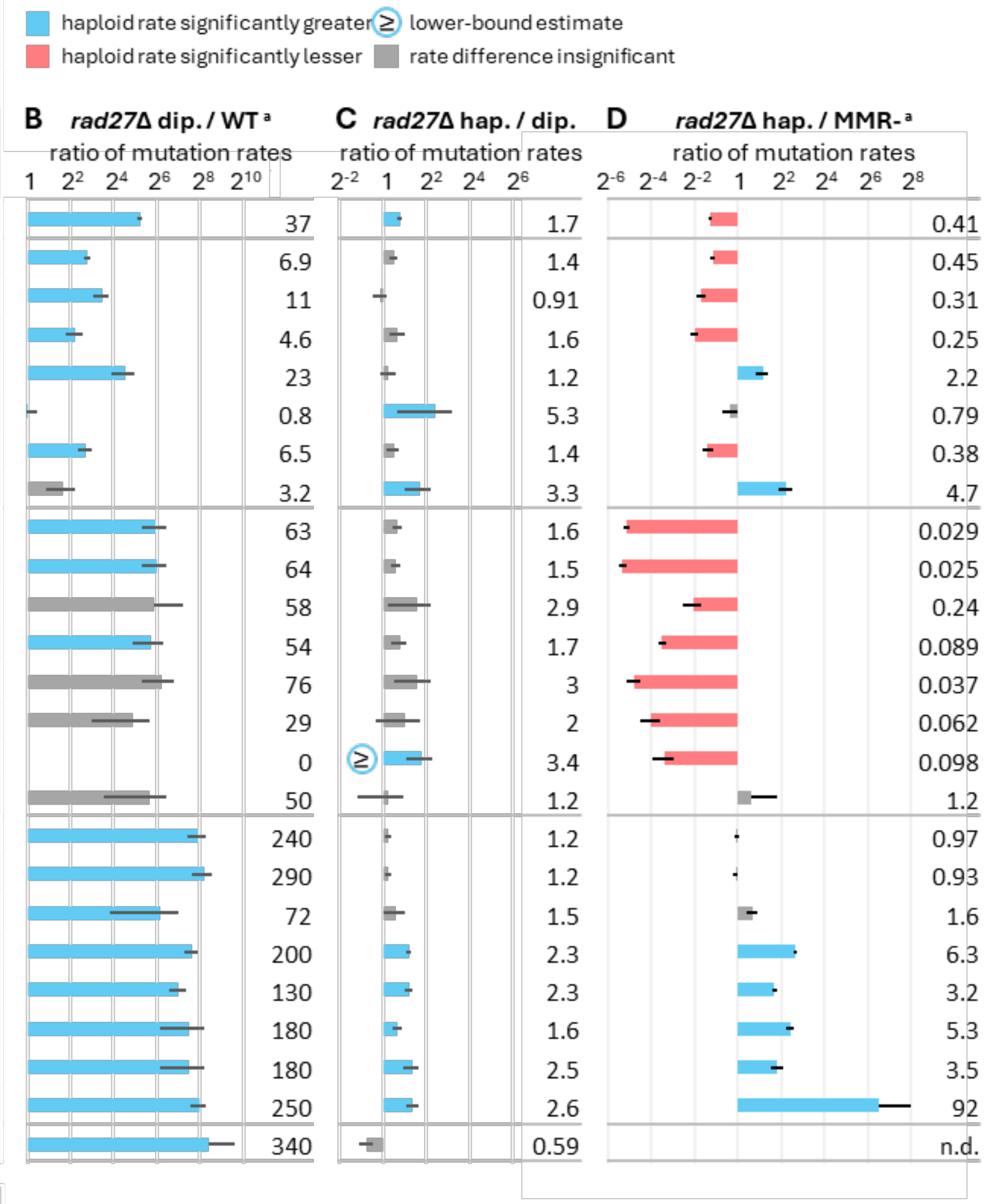

E	variant count		rate (Gbp^{-1} gen.$^{-1}$)		LOH
	definite	LOH	definite	LOH	(%)
**WT**	406	60	0.24	0.036	12.9
**MMR-**	21,966	2,936	37	4.9	11.8
***rad27Δ* diploid**	776	230	8.8	2.6	22.9

Supplementary Figure 4. Comparison of *rad27Δ* haploid and diploid mutation rates. (A) Mutation counts and rates. Notes: gen. = yeast generations; sterr. = standard error. (B-D) Rate ratios. Blue bars indicate significant increases, red bars significant decreases ($p < 0.0015$ for Šidák FWER = 0.05). Error bars depict standard errors. (B) Ratio of WT and diploid *rad27Δ* rates. (C) As per B but comparing rad27Δ haploid and diploid strains. Where no mutations were observed in the diploid set, lower bound ratios were estimated as if one mutation had been observed (≥). (D) Ratio of *rad27Δ* and MMR- rates, as per Figure 2. (E) Variant loci with loss of heterozygosity (LOH).

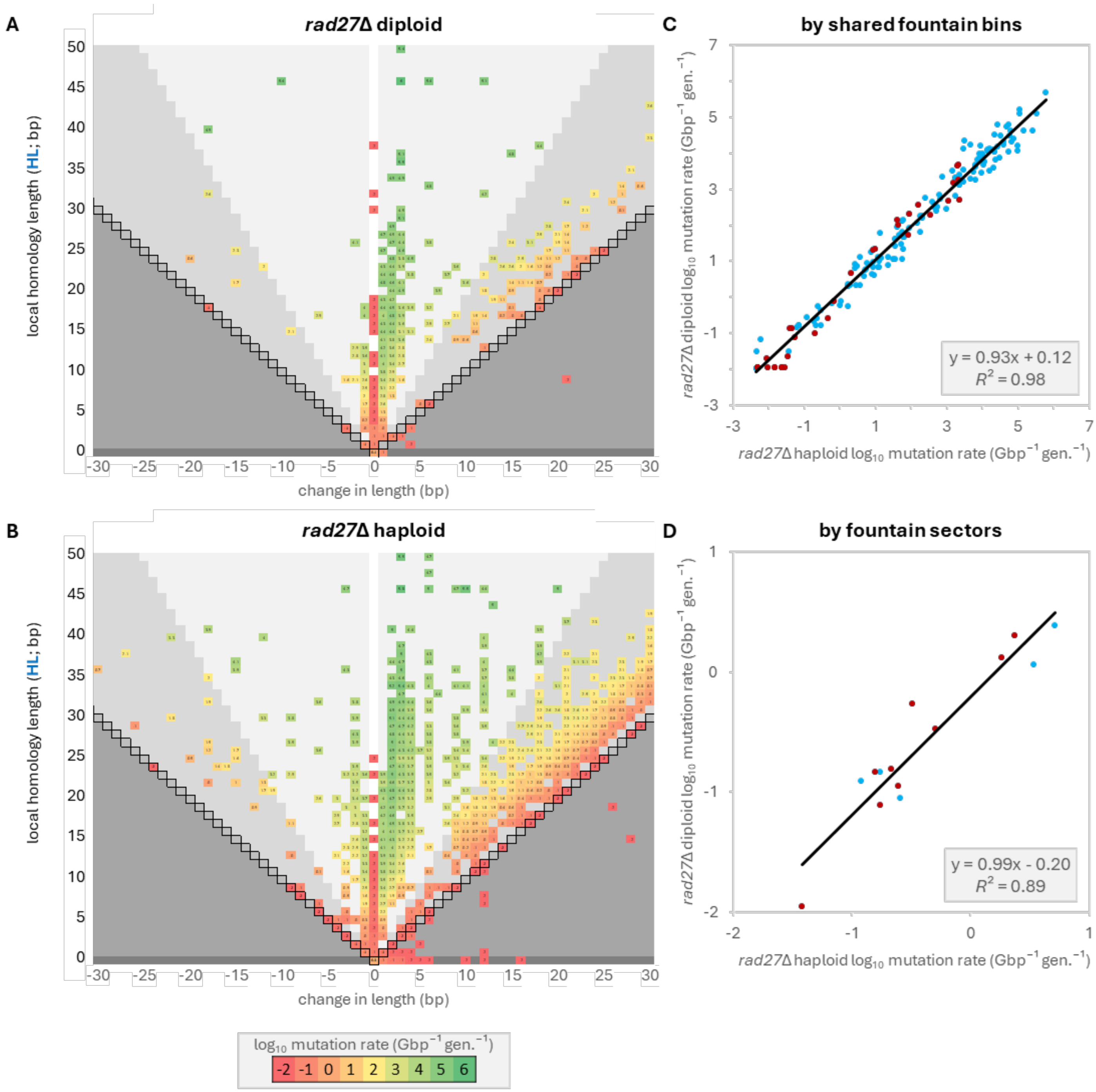

Supplementary Figure 5. Comparison of *rad27Δ* haploid and diploid mutation classes via fountain plots. (A-B) Fountain plots of *rad27Δ* diploid (A) and haploid (B) mutations. Diploid mutation patterns mimic haploid patterns, with greater sparsity caused by the smaller diploid collection. Rates are in log_{10}, per Gbp per generation. Fountain plot sectors are colored to match their corresponding mutation categories (Fig. 4). Deletions lie to the left (negative changes), insertions to the right, substitutions at the origin, and complex mutations along the y-axis. (C-D) Correlation between diploid and haploid rates on log-log plots. Points are colored by bin content, multi-base insertions in blue, all others in red. (C) Rates by fountain bin (count >0 in both collections). (D) Rates by fountain sector, with 1 bp A/T and G/C indels separated from sectors 4 and 7.

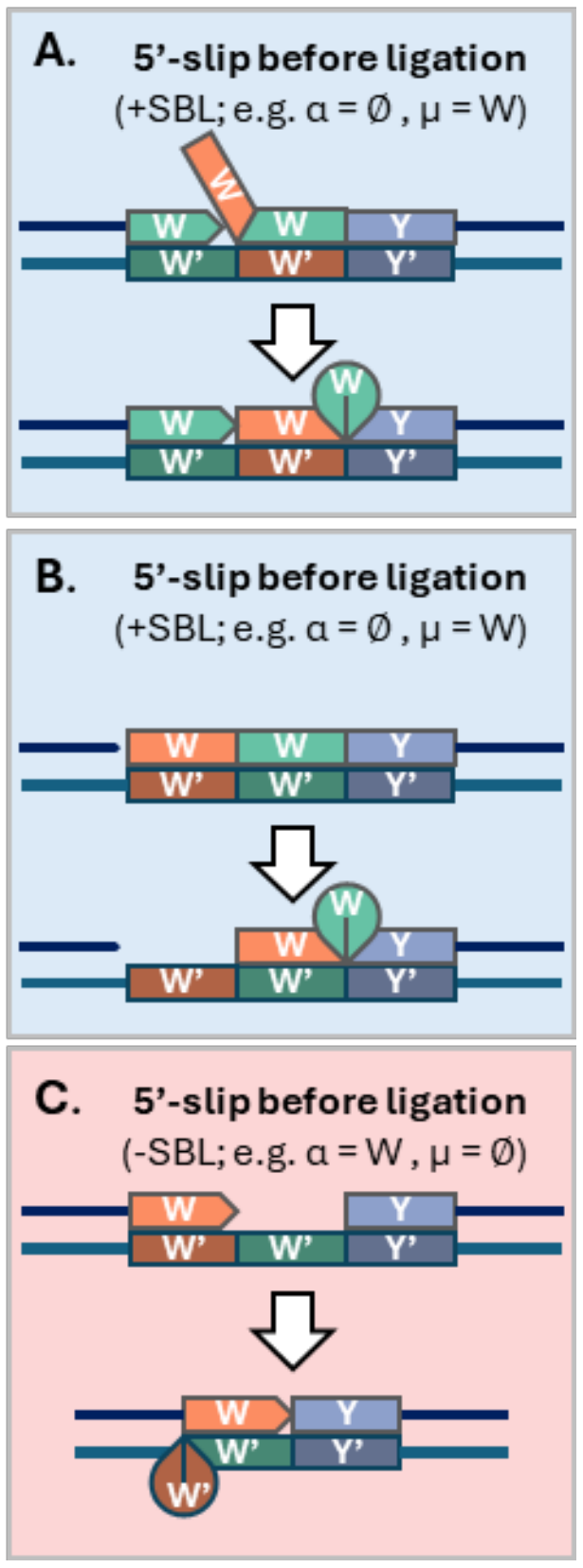

Supplementary Figure 6. Additional mutagenesis models. (A-C) Diagrams of strand slippage after replication but before ligation. Mechanisms that cause insertions (Ø → W or Ø → XW) or deletions (W → Ø or XW → Ø) are shown on blue and pink backgrounds, respectively. For simplicity, only the pathway steps that differentiate between mechanisms are shown. Notes: α = reference sequence; μ = mutant sequence; Ø = null, the absence of base pairs. If upstream template strand slippage makes room for (A) complementation of a 5'-flap or (B) patch resynthesis, then an insertion mutation will result in the next replication round (+SBL). (C) If the 5'-end of a primer strand slips forward to occupy unpaired template, then a deletion will occur (-SBL). This is similar to Streisinger slippage, except that it occurs after strand synthesis is completed.
