# Supplementary material for: Two distinct Okazaki fragment failure modes dominate mutagenesis in the absence of flap endonuclease 1
Source: Nat Commun. 2026 Jul 17;17:8749. doi: 10.1038/s41467-026-75267-3 (PMC13493898; doi:10.1038/s41467-026-75267-3)
Supplement: Supplementary file 2 [file 41467_2026_75267_MOESM2_ESM.pdf]

Description of Additional Supplementary Files

File Name: Supplementary Data 1

Description: A workbook containing 8 worksheets.

1. File_contents: a list of worksheets and their contents.
2. sample_list: a list of yeast whole genome mutation accumulation samples.
3. mutation_lists: lists of mutations by data set.
4. rad27_complexes: a list of complex mutations in the rad27Δ strain.
5. wt_CNVs: a table of CNVs by sample, local ploidy, and flanking features,
6. rad27_CNVs: a table of CNVs by sample, local ploidy, and flanking features.
7. msh2_CNVs: a table of CNVs by sample, local ploidy, and flanking features.
8. LOH: a table of point mutation & LOH counts and rates.
