# Supplementary material for: Two distinct Okazaki fragment failure modes dominate mutagenesis in the absence of flap endonuclease 1
Source: Nat Commun. 2026 Jul 17;17:8749. doi: 10.1038/s41467-026-75267-3 (PMC13493898; doi:10.1038/s41467-026-75267-3)
Supplement: Supplementary file 4 [file 41467_2026_75267_MOESM5_ESM.pdf]

nature portfolio

Peer Review File

Two distinct Okazaki fragment failure modes dominate mutagenesis in the absence of flap endonuclease 1

Corresponding Author: Dr Thomas Kunkel

Attachments originally included by the reviewers as part of their assessment can be found at the end of this file.

Version 0:

Reviewer comments:

Reviewer #1

(Remarks to the Author)
In this manuscript, Lujan et al performed deep, whole genome sequencing of yeast lacking RAD27, which encodes the flap endonuclease that has primary responsibility for Okazaki fragment maturation (OFM). Notably Rad27 also plays roles in a number of different repair pathways, with varying importance. The authors compared the mutation profile of rad27Δ strains with wild-type and mismatch repair- (MMR-) deficient strains. The authors identified a unique rad27Δ profile that included substitutions, insertions and deletion and copy number variation. They propose two distinct mechanistic models to account for these specific mutation profiles. The first is slippage before ligation (SBL), which they invoke as causing the majority of insertion mutations in rad27Δ cells. The second is transient primer relocation (TPR), which they propose accounts for substitutions, insertions, deletions and copy number variation, particularly mutations that involve non-adjacent templates. This work therefore uses a detailed analysis of sequence data to develop important models for the generation of mutations in the absence of RAD27 and provides context for the importance of RAD27 in promoting genome stability. Having said that, there are some questions about methodology. Further, previous work should be acknowledged and incorporated into the discussion of these results.

Major comments:
1. The comparison of the different genotypes is important. However, the authors compare haploid mutation profiles for rad27Δ with diploid mutation profiles for the wild-type and MMR- strains. It has been shown that there are significant differences in mutation rates and profiles in haploid and diploid strains, as well as differences in growth rate changes in mutation accumulation experiments (Serero et al, 2014 PNAS; Sharp et al, 2018 PNAS). This makes the comparisons less valid.
2. The authors note that “No serious growth defects were encountered with rad27Δ cells.” (lines 205-206). Was there any slow growth phenotype? It has been shown that rad27Δ has a slow-growth phenotype and loss of this phenotype could indicate second site suppressors. Were the strains used for the MA experiments MMS sensitive and was this retained throughout?
3. Distinct mutation profiles in MMR- and rad27Δ has been previously characterized and this should be more clearly acknowledged (e.g. Tishkoff et al, 1997 Cell; Xie et al 2001 MCB, Serero et al, 2014 PNAS as well as Omer et al, 2017 G3). Serero et al observed very similar mutation profiles as reported here. They also observed translocations and minisatellite instability. Similarly, the effects of rad27 deletions and mutations have been shown to be synergistic with defects in MMR, again indicating distinct pathways for mutagenesis, as argued by the authors in the discussion (e.g. Xie et al, 2001 MCB; Kumar et al , 2011 JMB).
4. Figure 4 was quite effective at highlighting the variability in mutation classes between genotypes.
5. The results section would be more effective if they were somewhat streamlined and clarified. The description of results jumps around a bit. The different classes of mutations are defined, starting on Line 489, after discussion of different types of mutations have already been discussed. The ICCs and ECCs discussion is a little confusing and the sections are not well defined (lines 454-487). The concept of local sequence homology is a little confusing. The examples given are useful, but the 1.25 consecutive copies on line 574 are unclear.
6. Important to note that mutation rates are not uniform throughout replication in wild-type cells (e.g. Lang & Murray, 2011 Genome Biol Evol). How is this affecting calculations and conclusions?
7. The discussion could also be streamlined and previous work can be referenced to support arguments., as well. Work from the Bambara lab would support the dynamic re-equilibration of 5’ flaps (e.g. Kao et al, 2022 JBC; Liu & Bambara, 2003 JBC; Kantartzis et al, 2012 Cell Rep). Work from the Kolodner lab provides nuance regarding he role of Rad27 in MMR (e.g.

Palacio et al, 2025 PNAS; Calil et al, 2012 Nat. Comm.).
8. The careful development of the SBL and TPR models are an important contribution. It would be useful to 1. Use published data where appropriate to support the models (see notes above) and 2. Propose genetic components that might be important for these proposed mechanisms. In some cases, there may already be sequence data available to analyze and correlate with the model predictions.

Minor comments:
1. The pathway models in Figure 1 could be abridged and simplified.
2. Figure 2F and the left side of Figure 5H are identical and could be consolidated – the data from the right side of Figure 5H could be moved or Figure 2F could be removed.
3. It's something of a semantic point, but lines 56/57 say "Such gaps resemble 5' flaps from the perspective of the enzyme." It might be more helpful for the reader to highlight the fact that the enzyme is recognizing the double-strand/single-strand junction with 5' ssDNA.
4. Overall, there is a dearth of references in the discussion in discussing potential pathways.
5. On lines 550-52, the authors suggested that stress related to loss of RAD17 could deplete dNTP pools late in replication. If anything, I would predict that this type of replication stress might increase dNTP pools. It is not analogous to addition of HU. And depleted dNTP pools would be predicted to lead to increased fork collapse and recombinational repair.
6. Rad27's role in BER is never considered in assessing the mechanisms underlying the mutation profiles, although NER is, in the context of TLS polymerases.

Reviewer #2

(Remarks to the Author)
This study by Lujan et al., performed whole-genome sequencing and mutation analysis in yeast strains lacking Rad27 and compared the mutation spectra as well as mutation rate with wild-type and MMR-deficient cells. They found that Rad27 mutant significantly increased mutation rate and complicated the mutational spectra. After comparing mutations in each category, the authors conclude that defects in Okazaki fragment maturation, including DNA terminus slippage before ligation (SBL) and transient primer relocation (TPR), are responsible for the observed mutational features in the Rad27 mutant. Overall, data presented in the study is solid and description of the results is straightforward.

The major concern is data interpretation and the underlying mechanisms. All the evidence comes from genome sequencing and mutation calling in yeast strains. The SBL and TPR models are solely based on mutation comparison between Rad27-deficient and proficient strains, and no other evidence is included. Mutation is the final outcome of defective cellular processes such as replication and repair. Although analyzing mutation spectra can provide important information, teasing out the mechanism requires complementary analysis of replication or repair in each background. But this study does not have such intermediate data and leaves an impression of data overinterpretation.

Some specific concerns:
1. Is there a replication-associated mutational strand asymmetry between leading and lagging strands in rad27 strain? This could provide some insights, particularly if the model points to defects in Okazaki fragment maturation.
2. The rad27 strain is a haploid but the other two reference strains (wt and MMR) are diploid. Is there a concern the ploidy difference affects mutation analysis?
3. The mutation rate calculation considers generations. However, as stated in the manuscript, the rad27 strain grows slower. How is the number of generations determined considering the differential doubling time?
4. There is an interesting observation of mutation peaks at the start of end of genes, as well as peaks around the replication origin. Authors suggested polyA/T nucleotides as the reason. However, it is clear that rad27 mainly has the peak at the end of the gene, but not the start. MMR deficient cells have two peaks (Fig 3, C and D). This doesn't support the data interpretation. Additionally, there is a high mutation peak overlapping the origin sequence in MMR deficient cells. But the peaks shift to left and right side of the midpoint of origin sequence in rad27 cells. The polyA/T sequence cannot explain the difference here either.
5. Figure 4 is too complicated.

Reviewer #3

(Remarks to the Author)
The Rad27/Fen1 nuclease has a well-documented role in flap removal during Okazaki fragment maturation (OFM). Additionally, the flap endonuclease activity of Rad27 is implicated in BER, RER and MMR mechanisms. Previous studies have shown a high rate of mutation accumulation in rad27 mutants and the spectrum of mutations, primarily insertions within repeat sequences, is consistent with aberrant processing of flaps during OFM. In this study, the authors performed deep sequencing of rad27 clones following a defined number of replication cycles and from this analysis conclude that insertional mutations are due to either DNA terminus slippage before ligation (SBL) or transient primer relocation (TPR), and are unlikely a result of defective MMR.

While the main conclusions of the paper are similar to other studies, the in depth analyses, including the role of flanking homologies, nucleotide sequence contribution and genomic sequence context, yield new insight into the mutational processes that occur in the absence of Rad27. The main concern I have with the data interpretation revolves around the use of a haploid rad27 strain whereas the wild-type and MMR-deficient strains used for comparison are all diploid. Since rad27

mutants have a strong mutator phenotype, the mutation rate could be under-estimated due to loss of function mutations in essential genes. Presumably, this is the reason why diploids of MMR-deficient strains were used in previous mutational studies by the authors as recessive lethal mutations are know to accumulate in the absence of MMR. Furthermore, Loeillet et al. (2019) identified large structural variants in the mutational spectrum of a rad27 diploid and these events would likely be missed when using haploid cells. The study by Loeillet et al reported a higher rate of mutation accumulation in rad27 cells than in msh2 cells, in contrast to what is reported here, and this disparity needs to be addressed by the authors. Although use of a rad27 diploid would be unlikely to change the main conclusion of the paper that SBL and TPR are the primary cause of mutations, the authors do need to explain their rationale for using a haploid rad27 strain and discuss the limitations in terms of the classes of mutations that might have been missed.

Minor comments:

1. Line 321: Since a haploid strain was used, how would LOH occur?
2. Line 440: Do CNVs detected in the rad27 strain have homologies at the breakpoints?
3. Line 449: Only whole chromosome gains could be detected in the rad27 haploid, whereas gains and losses can be detected in diploids.
4. Line 452: Persistent single-stranded breaks and gaps in rad27 strain are likely converted to double-strand breaks in the following S-phase, explaining the synthetic lethality between rad27 and HR mutations. HR repair of these lesions could be responsible for some of the structural variants detected in rad27 cells. Break-induced replication in sub-telomeric regions could contribute to CNVs involving chromosome ends. The authors should discuss the potential role of HR in some of the mutational events observed in rad27 cells.
5. Line 550: The replication stress observed in rad27 cells likely activates the replication and DNA damage checkpoints resulting in increased dNTP pools via Sml1 degradation.
6. Previous studies have concluded that the role of Rad27 in MMR is at best minor, and that the mutational spectrum of rad27 mutants is unique and distinct from MMR deficiency. Much of the Discussion is focused on the comparison between Rad27 loss and MMR deficiency, I felt this was unnecessary.

Version 1:

Reviewer comments:

Reviewer #1

(Remarks to the Author)
Overall, this revised version addresses my comments and the additional sequencing of the diploid strains is appreciated. A couple of minor points remain.
The extensive Figure 1 is useful in highlighting the different roles of Rad27/Fen1 in different DNA metabolism pathways. However, it overemphasizes the role that Rad27 plays or may play on certain pathways. For example, the role of Fen1 in some of the pathways described in Figure 1 are not well-established. For example, there is little evidence that Fen1/Rad287 is functionally important for nucleotide excision repair. Similarly, Fen1/Rad27 does nt play significant role in homologous recombination. It's role in the context of BRCA1/2 mutated cells was suggested to be related to a role in MMEJ. If the authors wish to retain the extensive figure, the description should be more nuanced.
In my view, the comparison of rad27Δ with HU treatment should be completely removed; it is a red herring. The remaining section of the modified results is appropriate. Further, I would suggest that rad27Δ-induced replication stress could be leading to altered origin usage, as has been shown for different types of replication stress. As the authors note, it also leads to differences in nucleosome positioning. These global changes complicate the comparison of replication timing with previous work done in the absence of replication stress and this caveat should be noted.
A minor point, one of the references to replication timing in Fig. 3 is for work performed in S. pombe. Muller and Nieduszynski, 2012, from S. cerevisiae might be more appropriate.

Reviewer #2

(Remarks to the Author)
The authors addressed my concerns with additional data and explanations. I support publication of the revised manuscript in Nature Communications.

Reviewer #3

(Remarks to the Author)
The authors have adequately addressed my concerns with the original submission.

Minor comments:
The authors have contradictory statements regarding growth of rad27 mutants. Line 204 states that rad27 haploids grow

about 50% slower than wild type, but line 212 states that no serious growth defects were encountered with rad27 cells. I suggest removing the sentence from lines 211-212 because a 50% growth defect is quite significant.

Line 785: The authors might consider adding Rad51 before nucleoprotein filament.

Open Access This Peer Review File is licensed under a Creative Commons Attribution 4.0 International License, which permits use, sharing, adaptation, distribution and reproduction in any medium or format, as long as you give appropriate credit to the original author(s) and the source, provide a link to the Creative Commons license, and indicate if changes were made.
In cases where reviewers are anonymous, credit should be given to 'Anonymous Referee' and the source.
The images or other third party material in this Peer Review File are included in the article's Creative Commons license, unless indicated otherwise in a credit line to the material. If material is not included in the article's Creative Commons license and your intended use is not permitted by statutory regulation or exceeds the permitted use, you will need to obtain permission directly from the copyright holder.
To view a copy of this license, visit https://creativecommons.org/licenses/by/4.0/

Reviewer 3's comments on synthetic lethality between *rad27*Δ and HR mutations led us to reckon with the similar mutagenic outcomes of homoeologous recombination and TPR. Here and in the manuscript you will find that we often refer to all mechanisms that result in segmental replacement through distal repriming collectively as template switching (TS). The relative merits of the different potential mechanisms are discussed.

All three reviewers called out the comparison of mutation rates from haploid *rad27*Δ strains with diploid WT and MMR- strains. To address this directly, we performed new mutation accumulation experiments in diploid *rad27*Δ strains. The results are summarized in new Supplemental Figures 4-5, three panels of which are excerpted below.

Figure S4, excerpted panel

A	*rad27Δ* haploid			*rad27Δ* diploid		
isolates	43			22		
total gen.	18,524			3,960		
mapped (Gbp)	0.49			0.49		
mutation type	count	rate (Gbp^{-1} gen.$^{-1}$)	rate sterr.	count	rate (Gbp^{-1} gen.$^{-1}$)	rate sterr.
all mutations	3,113	15	0.38	776	8.8	0.46
**substitutions**	383	1.9	0.096	117	1.4	0.13
A|T to G|C	75	0.58	0.057	35	0.64	0.13
G|C to A|T	87	1.1	0.12	23	0.69	0.15
A|T to T|A	84	0.65	0.078	30	0.55	0.11
A|T to C|G	25	0.20	0.038	2	0.037	0.025
G|C to T|A	66	0.83	0.13	21	0.63	0.093
G|C to C|G	46	0.58	0.079	6	0.18	0.064
**1 bp del.**	133	0.64	0.060	36	0.41	0.053
A|T del.	113	0.87	0.088	33	0.61	0.079
G|C del.	20	0.26	0.064	3	0.09	0.049
**> 1 bp del.**	100	0.48	0.066	25	0.29	0.058
2 bp del.	28	0.14	0.038	4	0.046	0.021
3 bp del.	14	0.067	0.02	3	0.034	0.019
4 bp del.	8	0.039	0.012	0	≤ 0.011	
> 4 bp del.	50	0.24	0.10	18	0.21	0.099
**1 bp ins.**	543	2.6	0.13	196	2.3	0.19
A|T ins.	490	3.8	0.20	181	3.3	0.29
G|C ins.	53	0.67	0.087	15	0.45	0.14
**> 1 bp ins.**	1,889	9.1	0.27	353	4.0	0.24
2 bp ins.	705	3.4	0.12	135	1.6	0.18
3 bp ins.	242	1.2	0.089	65	0.74	0.095
4 bp ins.	77	0.37	0.048	13	0.15	0.032
> 4 bp ins.	865	4.2	0.31	140	1.6	0.32
complexes	67	0.32	0.052	49	0.56	0.085

Figure S5, excerpted panel

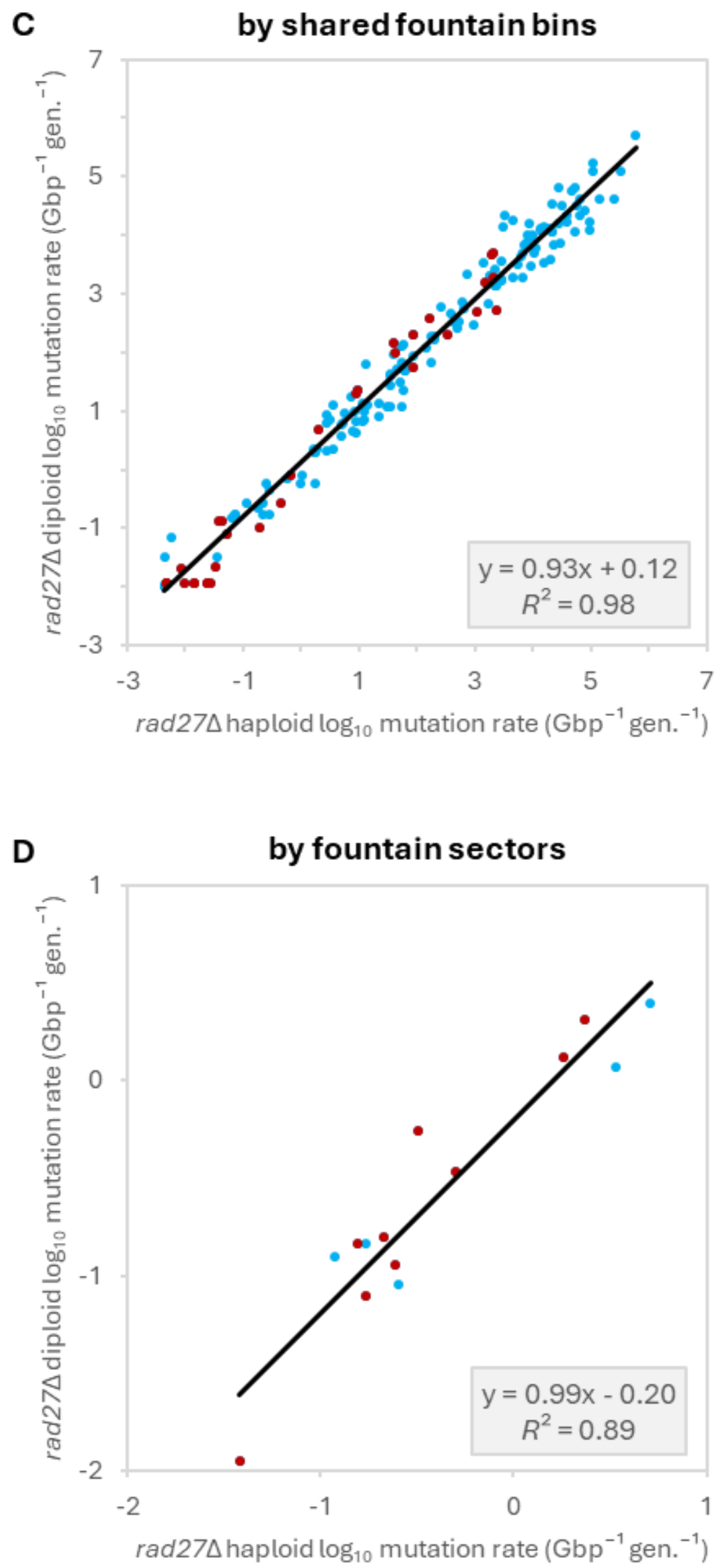

In short, we find that mutation rates and spectra of haploid and diploid *rad27Δ* cells are highly similar. These data indicate that the mutational processes described here are largely independent of ploidy. The differences do not materially change the primary conclusions of the paper but do preclude combining the haploid and diploid data sets. As such, we retain the haploid *rad27Δ* results in the main text and figures as it is the larger data set. We include diploid data in supplementary figures and address them in the Results and Discussion (see below for how they bolster the TS mechanisms).

In more detail, the following new section was added to the end of the Results:

"*The effects of rad27Δ are mildly affected by ploidy.*

Ploidy can affect mutation rates [42,109]. Overall mutations rates in the *rad27*Δ diploid were 1.7x lower than in the haploids, a small but highly significant difference (Fig. S4; $p < 2\times10^{-10}$; still higher than found in a previous study [99]). Most of the decrease (82%) appears to be attributable to a 2.3x decrease in multi-base insertion rates. However, closer inspection shows a consistent effect on most haploid and diploid indel rates, comparing mutations binned by mutation length and local homology length (Fig S6c, $R^2 = 0.98$) or by mutation type (Fig S6d, $R^2 = 0.89$). Discussion will focus on the former as it is the more robust data set (187 shared fountain plot bins vs. 14 shared fountain plot sectors with 1 bp indels treated separately from sectors 4 and 7). These are log-log plots in base ten, so a linear correlation with a slope near unity implies a uniform effect on the underlying mutational processes. A y-intercept of 0.12 means a 1.3x difference (i.e. $10^{0.12}$), also within error of unity. Though most shared bins are due to multi-base insertions, a uniform, mutation-type-independent effect is supported by the fact that none of the 32 deletion, substitution, 1 bp insertion, or complex mutation bins emerge as obvious outliers.

The 1.7x lower overall mutation rate in the *rad27*Δ diploid could be explained by a hypothetical misestimation of elapsed generation counts. However, a 1.7x change in cell divisions would mean only 17.6 generations per passage rather than 30, causing massive decrease in colony volume ($2^{30-17.3}$ = 5,200x, depending on growth assumptions). Large differences in generation counts are precluded by selection of colonies of predetermined size (see Methods). Strong selection could theoretically break the correlation between colony size and elapsed generations, but there is no obvious evidence of such selection. In fact, the slightly faster growing diploids, where selection should be weaker, had lower mutation rates."

Note that the previous study cited above is (Omer et al, 2017 G3). Most of the difference with their *rad27*Δ rates is attributable to higher indel rates in our strains. We attribute this mostly to the higher indel sensitivity of our muver mutation calling pipeline (PMC5944071), a point on which one author of that manuscript agreed (personal communication).

If the lower diploid rate is not explainable as experimental error, then why would increasing copy number decrease rates for nearly all mutation types? The only mechanism that we investigated that affects all mutation types is transient primer relocation (TPR). TPR only causes mutations when the two sites are heterologous. The diploid doubles the number of homologous sites, reducing the need for heterologous TPR. Neither Streisinger slippage, slippage before ligation, polymerase mispairing, looping during ligation, nor translesion synthesis rate should be affected by ploidy. Therefore, diploid rates bolster the case for TPR being a major mode of *rad27*Δ mutagenesis.

Based on the diploid results, a new Results section on LOH was added:

“214 LOH loci were detected in the *rad27*Δ diploid strain, compared to 727 mutations that were not caused by LOH (Fig. S5e). This represents a rate of 2.4 LOH loci generated per Gpb per generation. This is 69x the LOH rate of the WT set and about half the rate of the MMR- set. However, simple LOH rates are misleading when diploid strains begin with very little heterozygosity. If heterozygosity is driven by mutations accumulated after isogenic diploidization, then strains with higher mutation rates will have proportionally more heterozygosity and thus more opportunity for detection LOH. Instead, the fraction of variants attributable to LOH (LOH fraction), accounts for differences in heterozygous site density at the cost of measuring absolute rates. The diploid *rad27*Δ LOH fraction was 21.7%, almost twice that of WT and MMR- sets (12.7 and 11.8%, respectively).”

A new Discussion section was also added:
“How many TS events are invisible to mutation accumulation experiments because the primer relocates to a truly homologous locus? Note that the point mutation rate in the diploid *rad27*Δ strain is lower than in the haploid (Fig. S4a) but the LOH fraction is higher (Fig. S4e). This implies that increasing chromosome copy number increases accuracy, possibly by providing more opportunities for more homologous (invisible) or nearly homologous TS (LOH).”

Reviewer #1 (Remarks to the Author):

In this manuscript, Lujan et al performed deep, whole genome sequencing of yeast lacking RAD27, which encodes the flap endonuclease that has primary responsibility for Okazaki fragment maturation (OFM). Notably Rad27 also plays roles in a number of different repair pathways, with varying importance. The authors compared the mutation profile of rad27Δ strains with wild-type and mismatch repair- (MMR-) deficient strains. The authors identified a unique rad27Δ profile that included substitutions, insertions and deletion and copy number variation. They propose two distinct mechanistic models to account for these specific mutation profiles. The first is slippage before ligation (SBL), which they invoke as causing the majority of insertion mutations in rad27Δ cells. The second is transient primer relocation (TPR), which they propose accounts for substitutions, insertions, deletions and copy number variation, particularly mutations that involve non-adjacent templates. This work therefore uses a detailed analysis of sequence data to develop important models for the generation of mutations in the absence of RAD27 and provides context for the importance of RAD27 in promoting genome stability. Having said that, there are some questions about methodology. Further, previous work should be acknowledged and incorporated into the discussion of

these results.

Major comments:
1. The comparison of the different genotypes is important. However, the authors compare haploid mutation profiles for rad27Δ with diploid mutation profiles for the wild-type and MMR- strains. It has been shown that there are significant differences in mutation rates and profiles in haploid and diploid strains, as well as differences in growth rate changes in mutation accumulation experiments (Serero et al, 2014 PNAS; Sharp et al, 2018 PNAS). This makes the comparisons less valid.

Please see our discussion of diploid effects above. Serero and Sharp are cited therein.

2. The authors note that "No serious growth defects were encountered with rad27Δ cells." (lines 205-206). Was there any slow growth phenotype? It has been shown that rad27Δ has a slow-growth phenotype and loss of this phenotype could indicate second site suppressors. Were the strains used for the MA experiments MMS sensitive and was this retained throughout?

We thank the reviewer. The *rad27*Δ strain did exhibit a modest slow-growth phenotype consistent with prior reports, including our previous experience with haploids (PMID 33473124: Figs. 4a & S1b). No progressive growth defects were observed during mutation accumulation. The Methods now note "Haploid cells lacking *RAD27* grow about 50% slower than wild type [91], requiring an additional day of growth per passage, on average."

We did not systematically assay MMS sensitivity before, during, or after the MA experiment. However, the strain genotype and phenotype are consistent with previously characterized *rad27*Δ strains.

3. Distinct mutation profiles in MMR- and rad27Δ has been previously characterized and this should be more clearly acknowledged (e.g. Tishkoff et al, 1997 Cell; Xie et al 2001 MCB, Serero et al, 2014 PNAS as well as Omer et al, 2017 G3). Serero et al observed very similar mutation profiles as reported here. They also observed translocations and minisatellite instability. Similarly, the effects of rad27 deletions and mutations have been shown to be synergistic with defects in MMR, again indicating distinct pathways for mutagenesis, as argued by the authors in the discussion (e.g. Xie et al, 2001 MCB; Kumar et al , 2011 JMB).

We have added citations to Xie, Kumar, Serero, and Tishkoff to the Intro (section on Rad27 in MMR) and credited them with past comparisons between *rad27*Δ and MMR- data sets. Tishkoff was already cited in the first paragraph of the Intro. Omer was one of our touchstones, cited and directly compared to our results throughout.

4. Figure 4 was quite effective at highlighting the variability in mutation classes between genotypes.

We thank the reviewer for this kind comment.

5. The results section would be more effective if they were somewhat streamlined and clarified. The description of results jumps around a bit. The different classes of mutations are defined, starting on Line 489, after discussion of different types of mutations have already been discussed.

This was a matter of some debate among the authors before the initial submission. We needed to present the data binned into both familiar mutation types (Fig 1a), for consistency with previous publications, and into new mutation class (Fig 1f) compatible with fountain plotting (Fig 4). Leaving the latter until just before or even after introducing the fountains caused additional conclusion and redundancy. The consensus among the authors remains that both sets of definitions are needed and that changing the order will reintroduce old problems.

The ICCs and ECCs discussion is a little confusing and the sections are not well defined (lines 454-487).

These were defined in the methods, but not by exactly the same name, which was an error. Here is the corrected Methods section:

"Complex mutation candidates were defined as loci wherein multiple individually called mutations in one sample were discovered with separation of under 12 bp. The sequencing data were assessed manually and algorithmically to ensure that the component mutations were properly phased (found on the same sequencing read, and therefore on the same chromosome). Unfortunately, as mutation density increases in each outgrowth line, so does the probability that two independent mutations will occur close together. If the goal is to call mutation events with minimal false positives, then high mutation density precludes confident complex mutation calling. Complex mutations and their flanking sequences were compared to the reference genome via BLAST 95. Complex mutations were assigned as hairpin or pseudo-palindrome extensions of the mutation increased the length of a hairpin or pseudo-palindrome within 30 bp of the mutation loci. Complex mutations were assigned as segmental replacements if a sequence identical to both the mutant sequence and flanking sequence(s) was found elsewhere in the genome and the probability of finding such a good match was under 5%. In practice, approximately 12 matching nucleotides were required to establish statistical significance. Thus, shorter complexes require longer matching flanks to establish significance. If the best match was on the same chromosome, then the complex was assigned as an inter-chromosomal conversion (ICC). If all matching sequences were on other chromosomes, then the replacement was assigned as an

extra-chromosomal conversion (ECC). If equally good matches were found on the same and other chromosomes, the replacement was called ambiguous. If no good matches were found, the complex was tagged as "unknown"."

In addition, the first paragraph of the results section was split into two and the following explanation added:

"Complex mutations were categorized as hairpin/pseudo-palindrome extensions or as segmental replacements. Segmental replacements were further classified as inter-chromosomal conversions (ICCs), extra-chromosomal conversions (ECCs), or ambiguous conversions depending on whether the best matching donor sequence occurred on the same chromosome, different chromosome(s), or both. Complex mutations that matched no other loci in the genome with sufficient significance were classified as unknown."

The concept of local sequence homology is a little confusing. The examples given are useful, but the 1.25 consecutive copies on line 574 are unclear.

Corrected: "Example 2 is an ancestral sample with a 5′-ATTTA sequence (5 bp) at an outgrowth locus with the sequence 5′-ATTTATTTA. This is a 4 bp insertion of either ATTT or TTTA. Either way, the ancestral sequence represents 1.25 consecutive copies of the inserted sequence, a 4 bp increase in chromosome length with 5 bp of local homology."

6. Important to note that mutation rates are not uniform throughout replication in wild-type cells (e.g. Lang & Murray, 2011 Genome Biol Evol). How is this affecting calculations and conclusions?

Replication timing was addressed in Figure 3g-h and in the Results section under "Deleting *RAD27* preferentially increases mutation rates early in replication". This does not affect other rate calculations, for which the finest time unit is a whole cell division, nor does it affect the conclusions. As to the cause of decreasing rates vs. time in those cells, as stated in the Results "The mechanism behind these decreases is unknown. Perhaps stress due to *RAD27* deletion makes dNTP pools decrease with replication time, as seen for hydroxyurea treatment [104], and then favoring error proofreading over error extension."

7. The discussion could also be streamlined and previous work can be referenced to support arguments., as well. Work from the Bambara lab would support the dynamic re-equilibration of 5' flaps (e.g. Kao et al, 2022 JBC; Liu & Bambara, 2003 JBC; Kantartzis et al, 2012 Cell Rep). Work from the Kolodner lab provides nuance regarding he role of Rad27 in MMR (e.g. Palacio et al, 2025 PNAS; Calil et al, 2012 Nat. Comm.).

The following now introduces the Discussion section on SBL:

"Biochemical studies show that Fen1 acts on dynamic, equilibrating flap intermediates, including double-flap substrates, supporting the idea that unprocessed flaps can rearrange prior to ligation [16,112-114]."

The references are to Kao 2002 (rather than 2022), Liu 2003, Liu 2003, and Kantartzis 2012.

The following now introduces the Discussion section on MMR and RAD27:

"There is functional redundancy between the Exo1, Rad27, and Mlh1-Pms1 excision pathways during MMR [21,110] (Fig. 1e-f). RAD27 deletion could therefore only cause a partial MMR defect at best. We checked each mutation type for evidence of such defects."

8. The careful development of the SBL and TPR models are an important contribution. It would be useful to 1. Use published data where appropriate to support the models (see notes above) and 2. Propose genetic components that might be important for these proposed mechanisms. In some cases, there may already be sequence data available to analyze and correlate with the model predictions.

We have cited published data to support re-equilibration and SBL and to support the lack of a major MMR defect due to *RAD27* deletion (see above).

As for other genetic components that might illuminate these mechanisms, combining *RAD27* deletion with deletion of MutL homologues or *MSH3/6*, with mutator variants of the replicases, with altered Exo1 expression, or with ligase mutants (mutator, separation of function, and/or temperature sensitive) are the first that come to mind. Rad50-52, 54-55, 57, and 59 also come to mind, but these are synthetic lethal with *RAD27* deletion (see HR discussion with Reviewer 3). However, while small whole genome mutation accumulation data exists for a few these (e.g. Liu et al. 2025, PMID: 41051315), data sets of sufficient size and with ideal genetic combinations are lacking. Five members of our lab are developing several of these lines of inquiry.

Minor comments:

1. The pathway models in Figure 1 could be abridged and simplified.

Thank you for this suggestion, however we touch all the Rad27 roles outlined in that figure and feel it would be inadvisable to exclude any of them, for the sake of both curious readers and the folks who did the previous work.

2. Figure 2F and the left side of Figure 5H are identical and could be consolidated – the data from the right side of Figure 5H could be moved or Figure 2F could be removed.

We acknowledge the redundancy. However, readers of earlier drafts found placement in 2F essential for introducing the concepts and in 5H helpfully prevented flipping between

pages 15 and 29 when interpreting the rest of the data in 5H. Helpful ≠ essential, but removing the section of 5F will only leave a large block of white space. Therefore, we leave Figures 2 & 5 unaltered for now but are not opposed to removing the three columns of 5F if the editors so desire.

3. It's something of a semantic point, but lines 56/57 say "Such gaps resemble 5' flaps from the perspective of the enzyme." It might be more helpful for the reader to highlight the fact that the enzyme is recognizing the double-strand/single-strand junction with 5' ssDNA.

The new sentence reads: "Such gaps resemble 5′ flaps from the perspective of the enzyme, which recognizes the double-strand/single-strand junction with 5' ssDNA."

4. Overall, there is a dearth of references in the discussion in discussing potential pathways.

In the revised manuscript, there are now over 40 references in the Discussion, half of which were not cited in other sections.

5. On lines 550-52, the authors suggested that stress related to loss of RAD17 could deplete dNTP pools late in replication. If anything, I would predict that this type of replication stress might increase dNTP pools. It is not analogous to addition of HU. And depleted dNTP pools would be predicted to lead to increased fork collapse and recombinational repair.

We acknowledge that *RAD27* deletion and HU exposure are only analogous in as much as they are both stressors. In fact, as Reviewer 3 points out, *RAD27* deletion likely increases nucleotide pools (also see PMID: 28678842).

Nucleotide depletion is not a great hypothesis, but we felt compelled to add something beyond the previous line: "The mechanism behind these decreases is unknown." Any insights the reviewer could provide into why RAD27 deletion would cause higher insertion rates early in replication would be much appreciated.

The discussion of pools was moved from the Results to the final paragraph of the Discussion (future work), and expanded to:

"Additionally, an explanation is needed for the decrease in insertion rates in late replicating *rad27*Δ cells (Fig. 3i). Hypothetically, stress due to *RAD27* deletion makes dNTP pools decrease with replication time, as seen for hydroxyurea treatment [122], which would favor proofreading over error extension. However, evidence already exists that *RAD27* deletion increases dNTP pools via interactions between Rnr1, Dun1, and Sml1 [123]. Such pool increases should favor extension from erroneous structures and thus increase error rates. More work is needed."

6. Rad27's role in BER is never considered in assessing the mechanisms underlying the mutation profiles, although NER is, in the context of TLS polymerases.

Thank you for catching this. We included BER alongside NER when launching into the discussion of potential TLS but did not follow through in the following paragraph and defaulted to saying NER. This has been corrected, as the first line of the next paragraph now reads "Could the complex mutations in the *rad27*Δ strain reflect Pol ζ activity secondary to repair defects (BER, NER, etc.)?"

Reviewer #2 (Remarks to the Author):

This study by Lujan et al., performed whole-genome sequencing and mutation analysis in yeast strains lacking Rad27 and compared the mutation spectra as well as mutation rate with wild-type and MMR-deficient cells. They found that Rad27 mutant significantly increased mutation rate and complicated the mutational spectra. After comparing mutations in each category, the authors conclude that defects in Okazaki fragment maturation, including DNA terminus slippage before ligation (SBL) and transient primer relocation (TPR), are responsible for the observed mutational features in the Rad27 mutant. Overall, data presented in the study is solid and description of the results is straightforward.

The major concern is data interpretation and the underlying mechanisms. All the evidence comes from genome sequencing and mutation calling in yeast strains.

Please see our discussion of diploid effects above.

The SBL and TPR models are solely based on mutation comparison between Rad27-deficient and proficient strains, and no other evidence is included. Mutation is the final outcome of defective cellular processes such as replication and repair. Although analyzing mutation spectra can provide important information, teasing out the mechanism requires complementary analysis of replication or repair in each background. But this study does not have such intermediate data and leaves an impression of data overinterpretation.

We agree that mutation spectra represent endpoint outcomes. We have added discussion on HR as an (admittedly disfavored) alternative to TPR (see below) and have added the following caveats to the final paragraph of the paper:

"It should also be noted that mutation spectra in one organism are only one line of evidence required for proving a mutation mechanism. In vitro error profiles of purified components and structures with Rad27 bound to mutational intermediates would be supportive."

Some specific concerns:

1. Is there a replication-associated mutational strand asymmetry between leading and lagging strands in rad27 strain? This could provide some insights, particularly if the model points to defects in Okazaki fragment maturation.

We found no significant strand asymmetry by the identity of mutated bases. This suggests that the mutational processes are not strongly biased, as measured. However, this was unsurprising.

If the source of mutations was due to an MMR defect: wild type replicases impart very little substitution strand bias (as measures with MMR- strains; strongest bias for G/C to A/T, marginal, p-value 0.046).

If the source of mutations was strongly strand biased SBL or TS: these mechanisms are not expected to have strong sequence preferences that could leave a strand bias signal.

We hope to combine *RAD27* deletion with mutator polymerases in the future to look at strand asymmetry, among other things.

The strongest evidence that ties some mutations (i.e. multi-base insertions) to OFM is the way mutation rates peak directly adjacent to well-ordered nucleosome positions (discussed in response to concern #4 below).

2. The rad27 strain is a haploid but the other two reference strains (wt and MMR) are diploid. Is there a concern the ploidy difference affects mutation analysis?

Please see our discussion of diploid effects above.

3. The mutation rate calculation considers generations. However, as stated in the manuscript, the rad27 strain grows slower. How is the number of generations determined considering the differential doubling time?

Generation counts were estimated based on colony size at each bottleneck, as described previously. While *rad27*Δ cells grow somewhat more slowly, bottlenecking at defined colony size ensures comparable numbers of cell divisions per passage.

The following was added to the Methods:

"Haploid cells lacking *RAD27* grow about 50% slower than wild type [94], requiring an additional day of growth per passage, on average. Growing colonies to the same size ensures a similar number of elapsed cellular generations, assuming similar cell volumes and rates of cell death."

4. There is an interesting observation of mutation peaks at the start of end of genes, as well as peaks around the replication origin. Authors suggested polyA/T nucleotides as

the reason. However, it is clear that rad27 mainly has the peak at the end of the gene, but not the start. MMR deficient cells have two peaks (Fig 3, C and D). This doesn't support the data interpretation. Additionally, there is a high mutation peak overlapping the origin sequence in MMR deficient cells. But the peaks shift to left and right side of the midpoint of origin sequence in rad27 cells. The polyA/T sequence cannot explain the difference here either.

We agree that polyA/T content alone does not fully explain all of these patterns. In fact, we state in the Results section entitled "Deleting RAD27 preferentially increases indel rates at loci with high local homology" that "higher indel rates in long tracts … explain neither the twin multi-base insertion peaks at origins (Fig. 3e) nor higher indel rates in early replication (Fig. 3g) seen in *rad27*Δ yeast." Nor do they explain why the 5/3' UTR multi-base insertion peaks hug the first/last nucleosomes.

In the Discussion, we rationalize the multi-base peak positions thusly:

"If these indels, primarily multi-base insertions, are indeed caused by defects in OFM, then they should be concentrated where OFM is most common, for example at replication origins. We previously used ribonucleotide incorporation during nuclear DNA replication [27] to test a proposal by O'Donnell and colleagues [112] that replication of both the leading and lagging DNA strands is initiated at replication origins by synthesis of an Okazaki fragment. When mapped against the whole genome, multi-base insertion rates peak on either side of replication origins (Fig. 3e). The centroids of these peaks are, on average, just on the origin side of the +1 and -1 nucleosomes, as was previously observed for Rad27p-cleaved OF termini [113]. This strongly suggests that some of the *rad27*Δ multi-base insertions are associated with OF termini."

5. Figure 4 is too complicated.

We agree that Figure 4 (and several others) are quite complicated. There is little we can do about simplifying individual graphs without discarding useful information, but we have clarified what each graph represents, and which graphs should be compared, by

- labelling panel sets by which genomic feature anchors the graph,
- adding space between rows of panels to decrease visual density,
- adjusting panel labels (A, B, etc.) to increase their visibility,
- and adding feature (ORF) diagrams to panels A-D.

Reviewer #3 (Remarks to the Author):

The Rad27/Fen1 nuclease has a well-documented role in flap removal during Okazaki fragment maturation (OFM). Additionally, the flap endonuclease activity of Rad27 is

implicated in BER, RER and MMR mechanisms. Previous studies have shown a high rate of mutation accumulation in rad27 mutants and the spectrum of mutations, primarily insertions within repeat sequences, is consistent with aberrant processing of flaps during OFM. In this study, the authors performed deep sequencing of rad27 clones following a defined number of replication cycles and from this analysis conclude that insertional mutations are due to either DNA terminus slippage before ligation (SBL) or transient primer relocation (TPR), and are unlikely a result of defective MMR.

While the main conclusions of the paper are similar to other studies, the in depth analyses, including the role of flanking homologies, nucleotide sequence contribution and genomic sequence context, yield new insight into the mutational processes that occur in the absence of Rad27. The main concern I have with the data interpretation revolves around the use of a haploid rad27 strain whereas the wild-type and MMR-deficient strains used for comparison are all diploid. Since rad27 mutants have a strong mutator phenotype, the mutation rate could be under-estimated due to loss of function mutations in essential genes. Presumably, this is the reason why diploids of MMR-deficient strains were used in previous mutational studies by the authors as recessive lethal mutations are know to accumulate in the absence of MMR. Furthermore, Loeillet et al. (2019) identified large structural variants in the mutational spectrum of a rad27 diploid and these events would likely be missed when using haploid cells. The study by Loeillet et al reported a higher rate of mutation accumulation in rad27 cells than in msh2 cells, in contrast to what is reported here, and this disparity needs to be addressed by the authors. Although use of a rad27 diploid would be unlikely to change the main conclusion of the paper that SBL and TPR are the primary cause of mutations, the authors do need to explain their rationale for using a haploid rad27 strain and discuss the limitations in terms of the classes of mutations that might have been missed.

We agree that ploidy is an important consideration and have conducted additional experiments to address it. Please see our discussion of diploid effects above.

Loeillet et al. found that overall *msh2*Δ error rates (10.55 per clone per passage) were actually 5x higher than *rad27*Δ rates (2.07 per clone per passage), but perhaps the reviewer was referring specifically to rates of structural variants? If so, Loeillet et al. found 0.04 CNVs per clone per passage, somewhat higher than the 0.006 for our haploid *rad27*Δ strain. Unfortunately, in our experience, fully automated CNV validation leads to high false positive rates, and we could not manually validate diploid *rad27*Δ CNVs in time for this resubmission. We will include them in an upcoming paper on *RAD27* deletion in the presence and absence of mutator replicase variants.

Minor comments:
1. Line 321: Since a haploid strain was used, how would LOH occur?

We think the reviewer is referring to the 70 cases of “LOH” mentioned at the beginning of the results. Thank you for catching this. The haploid rad27 cells had a very high CNV rate (19x wt; 4.3x MMR-). The 70 variants were all caused by combining point mutations with CNVs, creating loci the look like LOH (n > 1, >1 identical non-reference bases).

The corrected text:

“Another 70 variants were caused by combining point mutations with CNVs, discussed below regarding loss of heterozygosity (LOH). Another 12 variants were attributed to subclonal insertions in ancestral samples. These 82 variants are not included in mutation counts but may be found in Supplemental File 1.”

A paragraph was also added in the new Results section on LOH:
“Some loss of heterozygosity (LOH), wherein a heterozygous locus becomes homozygous, was already detectable in the *rad27*Δ haploids via combined CNVs and point mutations. Three point mutations occurred in CNV loci during the experiment, followed by LOH later in the experiment. Twelve more occurred in CNV loci before the experiment, followed by LOH during the experiment. 55 point mutations that predated the experiment and were then caught in CNVs during the experiment. Given the small fraction of mapped base pairs involved in positive CNVs, this represents a high LOH rate.”

Note that template switching, via TPR or homoeologous recombination, can cause LOH (see Discussion on diploid data above).

2. Line 440: Do CNVs detected in the rad27 strain have homologies at the breakpoints?

Yes. The following was added to the Results section on CNVs:
“In both *msh2*Δ and *rad27*Δ strains the remaining CNVs, those not involved in aneuploidy or chromosome ends, terminate on homologous loci. These were usually pairs of transposons, orphaned LTRs, or paralogous genes (Supplemental File 1).”

3. Line 449: Only whole chromosome gains could be detected in the rad27 haploid, whereas gains and losses can be detected in diploids.

The reviewer is correct. Please see our discussion of diploid effects above.

4. Line 452: Persistent single-stranded breaks and gaps in rad27 strain are likely converted to double-strand breaks in the following S-phase, explaining the synthetic lethality between rad27 and HR mutations. HR repair of these lesions could be responsible for some of the structural variants detected in rad27 cells. Break-induced replication in sub-telomeric regions could contribute to CNVs involving chromosome ends. The authors should discuss the potential role of HR in some of the mutational events observed in rad27 cells.

This was an oversight on our part. In fact, TPR and defective HR (homoeologous recombination) are difficult to distinguish with the current data. The literature surrounding synthetic lethality between *rad27*Δ and HR mutations forced us to reckon with how little we had considered homoeologous recombination. The Abstract and large segments of the Discussion, including Fig 2, have been rewritten. We now refer to TPR as one mutagenic template switching (TS) mechanism.

For example, in the Discussion:

"*Template switching is consistent with many rad27*Δ *mutations.*

A 3′-primer terminus that dissociates from its template can relocate to an alternative template (Fig. 5g). Following some amount of synthesis, a second relocation event could return the new nascent end to original template. Such template switching (TS) could cause segmental replacements either during (S phase) or outside of normal replication (G2 or late S phase).

During replication, transient primer relocation (TPR) is an extreme form of transiently initiated mutagenesis (TIM [88]). TPR requires the relocation of a 3′-primer terminus, but 5′-flaps are the structure on which Fen1 most famously works. Why would unpaired 3′-flaps arise? The ideal substrate for yeast Fen1 is actually a dually flapped substrate [115]. This could be created via partial re-equilibration of 5′-flap, which would disrupt base pairs on the opposite terminus. While yeast Fen1 prefers a substrate with a 1 bp 3′-flap and a longer 5′-flap, runaway re-equilibration could create extensive 3′-flaps in the absence of proper 5′-flap processing.

Homologous recombination (HR) involves the coordinated action of many proteins for strand invasion and resolution (reviewed [110]). Homoeologous recombination is an error-prone mode of HR involving similar but not identical DNA sequences. HR most commonly occurs during either meiotic crossover or during double strand break repair (DSBR). Constant nutrient abundance during mutation accumulation experiments encourages vegetative reproduction over meiosis and its associated recombination. The most likely candidate would be homoeologous recombination due to erroneous synthesis-dependent strand annealing (SDSA), (reviewed [110]). SDSA could be thought of as TPR with more steps, such as nucleoprotein filament formation (requiring Rad51 and/or Dmc1), strand invasion, and junction resolution. Notably, HR is absolutely required for viable haploid cells in the absence of Rad27 activity, which was attributed to repair of frequent double-stranded breaks [125,126]. Previous substitution spectra from *rad27*Δ cells also have high cosine similarity to spectra from cells with individual deletions of several HR components including RAD51 [127]."

Later in the Discussion:

“More work is required to distinguish between alternative TS mechanisms. In the meantime, we provisionally favor replication-associated TPR as a major contributor to TS events in *rad27*Δ cells. Notably, these cells still retain active MMR components such as MutSα, which suppress recombination between diverged sequences (homoeologous recombination; reviewed 110). Diploid *rad27*Δ cells have lower mutation rates (Fig. S4a,c), yet show a higher fraction of loss-of-heterozygosity (LOH) events relative to MMR-deficient strains (Fig. S4e). This suggests rampant rad27Δ TS events even when homoeologous recombination should be repressed. Distinguishing between replication- and recombination-associated TS will require future experiments, for example by defining the timing of TS events during S phase.”

We would characterize the need for such future experiments as urgent but beyond the scope of this study.

5. Line 550: The replication stress observed in rad27 cells likely activates the replication and DNA damage checkpoints resulting in increased dNTP pools via Sml1 degradation.

Please see our response to Reviewer 2, Minor Comment 5, for changes acknowledging this fact.

6. Previous studies have concluded that the role of Rad27 in MMR is at best minor, and that the mutational spectrum of rad27 mutants is unique and distinct from MMR deficiency. Much of the Discussion is focused on the comparison between Rad27 loss and MMR deficiency, I felt this was unnecessary.

The authors must disagree, as we entered the experiments thinking that, given a sufficiently large mutation spectrum, any MMR defect would dominate certain classes of mutations even if Fen1-dependent MMR is a small fraction of total MMR. The data do not support this initial hypothesis, which we consider notable enough to bear repeating for each mutation class.

Reviewer #1 (Remarks to the Author):

Overall, this revised version addresses my comments and the additional sequencing of the
diploid strains is appreciated.

Thank you.

A couple of minor points remain.

The extensive Figure 1 is useful in highlighting the different roles of Rad27/Fen1 in different
DNA metabolism pathways. However, it overemphasizes the role that Rad27 plays or may play on certain pathways. For example, the role of Fen1 in some of the pathways described in Figure 1 are not well-established. For example, there is little evidence that Fen1/Rad287 is functionally important for nucleotide excision repair. Similarly, Fen1/Rad27 does nt play significant role in homologous recombination. It's role in the context of BRCA1/2 mutated cells was suggested to be related to a role in MMEJ. If the authors wish to retain the extensive figure, the description should be more nuanced.

The roles for Fen1 in HR and NER were reported in the cited papers so we have chosen to preserve the current figure. However, we agree that the evidence therein was not necessarily conclusive. Therefore, we made the following textual changes (changes in bold and underlined):

1. in the Figure 1 legend, "Fen1, and sometimes Dna2, **<u>may process</u>** flaps after homologous recombination, microhomology-mediated end joining and nucleotide excision repair"; and
2. on page 5, "Homologous recombination (HR) [16], microhomology-mediated end joining (MMEJ) [11,16,73], and nucleotide excision repair (NER) [74,75] all generate flaps that **<u>may be processed</u>** by Fen1, with or without Dna2, depending on flap length and RPA binding (Fig. 1m**<u>; strength of evidence varies</u>**)."

In my view, the comparison of rad27Δ with HU treatment should be completely removed; it is a red herring. The remaining section of the modified results is appropriate. Further, I would suggest that rad27Δ-induced replication stress could be leading to altered origin usage, as has been shown for different types of replication stress. As the authors note, it also leads to differences in nucleosome positioning. These global changes complicate the comparison of replication timing with previous work done in the absence of replication stress and this caveat should be noted.

The statement on HU treatment was removed and the final sentence of the penultimate paragraph now reads "More work is needed to resolve this contradiction and to

determine whether rad27Δ-induced replication stress alters origin usage and replication timing."

A minor point, one of the references to replication timing in Fig. 3 is for work performed in *S.*
pombe. Muller and Nieduszynski, 2012, from *S. cerevisiae* might be more appropriate.

Thank you for catching this error. The mapping used was from Muller et. al and the reference has been replaced. The Methods also no directs readers to the study in which we described the the creation of the timing maps: "Replication time and nucleosome density maps we reported previously [83]."

Reviewer #2 (Remarks to the Author):

The authors addressed my concerns with additional data and explanations. I support publication of the revised manuscript in Nature Communications.

Thank you.

Reviewer #3 (Remarks to the Author):

The authors have adequately addressed my concerns with the original submission.

Thank you.

Minor comments:
The authors have contradictory statements regarding growth of rad27 mutants. Line 204 states that rad27 haploids grow about 50% slower than wild type, but line 212 states that no serious growth defects were encountered with rad27 cells. I suggest removing the sentence from lines 211-212 because a 50% growth defect is quite significant.

The use of "significant" in the sentence in lines 211-212 was improper. That sentence and the preceding one now read as follows:
"If, before the last intended passage, a prohibitive growth defect was acquired, then the sample from the last pre-defect passage was used instead. No prohibitive growth defects were encountered with *rad27*Δ cells."

Line 785: The authors might consider adding Rad51 before nucleoprotein filament.

Done, there and in the Figure 5 legend.
